# Efficient Sunlight Harvesting by A_4_ β-Pyrrolic Substituted Zn^II^ Porphyrins: A Mini-Review

**DOI:** 10.3389/fchem.2019.00177

**Published:** 2019-04-11

**Authors:** Gabriele Di Carlo, Alessio Orbelli Biroli, Maddalena Pizzotti, Francesca Tessore

**Affiliations:** ^1^Dipartimento di Chimica, Università degli Studi di Milano, UdR INSTM Milano, Milan, Italy; ^2^Istituto di Scienze e Tecnologie Molecolari del CNR (CNR-ISTM), SmartMatLab Centre, Milan, Italy

**Keywords:** solar energy, dye-sensitized solar cells, porphyrin-sensitized solar cells, porphyrins, light harvesting

## Abstract

Dye-Sensitized Solar Cells (DSSCs) are a highly promising alternative to conventional photovoltaic silicon-based devices, due to the potential low cost and the interesting conversion efficiencies. A key-role is played by the dye, and porphyrin sensitizers have drawn great interest because of their excellent light harvesting properties mimicking photosynthesis. Indeed, porphyrins are characterized by strong electronic absorption bands in the visible region up to the near infrared and by long-lived π^*^ singlet excited states. Moreover, the presence of four *meso* and eight β-pyrrolic positions allows a fine tuning of their photoelectrochemical properties through structural modification. *Trans*-A_2_BC push–pull Zn^II^ porphyrins, characterized by a strong and directional electron excitation process along the push–pull system, have been extensively investigated. On the other hand, A_4_ β-pyrrolic substituted tetraaryl Zn^II^ porphyrins, which incorporate a tetraaryl porphyrinic core as a starting material, have received lower attention, even if they are synthetically more attractive and show several advantages such as a more sterically hindered architecture and enhanced solubility in most common organic solvents. The present contribution intends to review the most prominent A_4_ β-substituted Zn^II^ porphyrins reported in the literature so far for application in DSSCs, focusing on the strategies employed to enhance the light harvesting capability of the dye and on a comparison with *meso*-substituted analogs.

## Introduction

Since the first appearance of the revolutionary work by Grätzel in 1991 (O'Regan and Grätzel, 1991), Dye Sensitized Solar Cells (DSSCs) emerged as a cutting-edge technology in the field of photovoltaics (PV).

**Figure 1:**
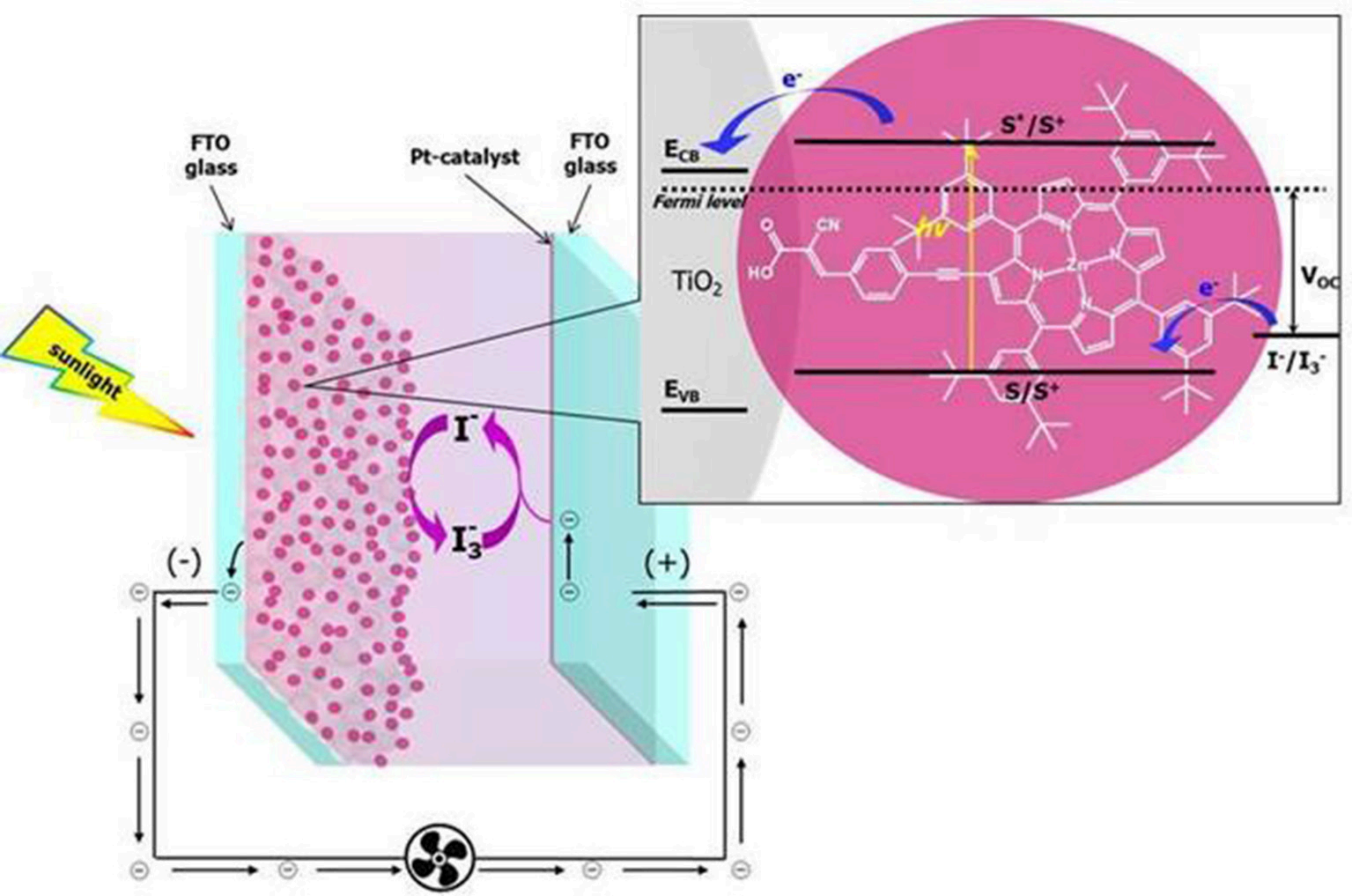
Working principle of a dye-sensitized solar cell.

DSSCs belong to the third generation PV technology and they consist of a nanocrystalline and mesoporous high band-gap semiconductor (usually TiO_2_), an organometallic or organic dye covalently linked to its surface and an electrolyte comprising a redox shuttle (typically I^−^/${\text{I}}_{3}^{-}$ or Co^III^/Co^II^). Often DSSC devices are comprised in the organic photovoltaic (OPV) family due to the organic nature of the dyes. In conventional OPV devices, donor and acceptor organic materials are blended and act as light absorbers and charge carriers simultaneously. On the other hand, in DSSCs, the light harvesting task is mainly deputed to the dyes anchored to the semiconductor which in turn serves, along with the electrolyte, as charge transporting material (Yum et al., 2011). Consequently, in this last technology, the light capture ability and the charge transport dynamics can be tuned separately, acting respectively on a rational tailoring of the dye, thus affecting its spectral response, or on the carrier transport properties of the semiconductor and the electrolyte. The light absorption by the anchored dye promotes the photoexcitation of electrons from the ground to the excited state orbitals followed by the electron-injection into the conduction band (CB) of the semiconductor. The injected electrons diffuse through the semiconductor layer toward the FTO transparent conducting glass, to reach a platinum counter electrode through the external wiring. Finally, the electrons are transferred to the redox shuttle which, in turn, regenerates the oxidized dyes, thus completing the electrical circuit (Figure 1).

Nowadays, DSSCs are considered a complementary option rather than as an alternative to the widely used silicon-based solar cells, due to the different market targeting in which they are involved. Indeed DSSCs, thanks to their transparency and their capacity to absorb diffuse sunlight, are very promising for the fabrication of PV windows and façades in the Building Integrated Photovoltaics (BIPV) (Di Carlo et al., 2018) and for indoor applications (Freitag et al., 2017). On the contrary, silicon solar cells don't allow the light to pass through, and are featured by a dark-blue standard color and can be installed only onto the rooftop of buildings or on the ground, despite the fact that they can rely on higher efficiencies and a broader spectral response.

The performance of a solar cell is strictly related to the ability of a the device to absorb and convert sunlight into electricity and it is evaluated by the overall solar-to-energy conversion efficiency (PCE) (Equation 1):

(1) $$\begin{array}{r} PCE=\frac{J_{sc}\;V_{oc}\;FF\;}{P_{in}} \end{array}$$

where *J*_*sc*_ is the short-circuit photocurrent density (A cm^−2^), *V*_*oc*_ is the open-circuit photovoltage (V), *FF* is the fill factor (that can assume values between 0 and 1) and *P*_*in*_ is the intensity of the incident wavelength (W cm^−2^).

Another very important parameter of the solar cell is the incident photon-to-current conversion efficiency (IPCE). The IPCE is a function of the wavelength and is expressed by Equation 2:

(2) $$\begin{array}{r} IPCE\left(\lambda \right)=LHE\left(\lambda \right)\;\phi_{inj}\left(\lambda \right){\;\varphi }_{reg}\left(\lambda \right){\;\eta }_{coll} \end{array}$$

where LHE is the light harvesting efficiency for photons of wavelength λ, ϕ_*inj*_ is the quantum yield for the injection of the photogenerated electron in the CB of the semiconductor, ϕ_*reg*_ is the quantum yield for dye regeneration and η_*coll*_ is the electron collection efficiency.

The LHE is related to the absorbance A of the sensitized semiconductor film (Equation 3):

(3) $$\begin{array}{r} LHE\left(\lambda \right)=1-10^{-A} \end{array}$$

Conventional DSSCs exhibit only a partial overlap of their own absorption spectra with the wide range of wavelengths of the solar spectrum, with a poor response to red and near-infrared (NIR) light, thus encouraging the scientific community to devote great efforts in developing a number of ways to overcome this limitation.

The investigated strategies to improve the IPCE of DSSCs mainly involve the fabrication process of the device by acting on its several components. For example, increasing the thickness of the TiO_2_ layer provides a concomitant increase of the chromophores concentration adsorbed into the semiconductor which, in turn, enables the improvement of spectral response intensity (Kao et al., 2009).

Additives, as highly luminescent energy-relay dyes (ERDs) in the electrolyte solution or anchored onto TiO_2_, enhance the conversion efficiency by non-radiative Förster-type excitation energy transfer or by radiative-type fluorescent energy transfer to the sensitizers (Rahman et al., 2015).

In order to also collect the incident photons in the range of wavelengths poorly adsorbed by the sensitizers, luminescent spectral conversion approaches have also been explored, ranging from down-shifting (DS) to up-conversion (UC). In the former case, materials enabling the conversion of high-energy photons (i.e., UV light) into lower-energy ones (i.e., Visible light) are used; the opposite happens in the latter case (Llanos et al., 2018).

All the above listed strategies are effective enough, however, they make the manufacturing process of the devices far from trivial, limiting the industrial application.

Hence, the most promising approach to enhance the solar light harvesting by DSSCs is the improvement of the light-capture ability of the dye over the entire visible and near-infrared (NIR) spectrum, by a rational tailoring of the structure, the electronic absorption spectrum, the molar extinction coefficient and the loading onto the semiconductor surface.

Since the accessible anchoring sites on the semiconductor are limited, the dyes must be well-chosen, taking into account the loading capacity of each chromophore. Furthermore, a large molar extinction coefficient of the dyes is required to get intense spectral response, still maintaining a thin film of TiO_2_. Co-sensitization of the semiconductor surface with different dyes having complementary absorption bands has also been developed to obtain a panchromatic light harvesting (Song et al., 2018).

The final aim is obviously the fabrication of an efficient device, able to cover the greatest part of the solar spectrum, while still ensuring intense electronic absorption. Indeed, the final device should absorb ~80% of the solar spectrum (from 350 to 900 nm) for commercialization.

Since 1993, a leading role among sensitizers has been played by Ru^II^ complexes, with efficiencies up to 11% (Nazeeruddin et al., 1993, 2005; Yu et al., 2010). However, the high cost and the limited supply of ruthenium, together with the poor absorption of these dyes in the NIR range of wavelengths, limited their widespread application.

On the other hand, metal-free organic sensitizers are advantageous because of their low cost, easy synthesis and flexible functionalization, and power efficiencies of 14% have been reached (Kakiage et al., 2015).

In addition to the aforementioned classes of sensitizers, porphyrin dyes have emerged as a viable and interesting alternative. Indeed, porphyrins satisfy the principal requirements for efficient solar energy collection. They possess strong electronic absorption bands (the B or Soret band around 400–450 nm and the Q bands in the range 550–650 nm, with molar extinction coefficients typically over 100,000 M^−1^ cm^−1^), and long-lived π^*^ excited states of appropriate energy to allow the injection of the photogenerated electron in the CB band of TiO_2_. Moreover, their many different reaction sites (namely four *meso*, eight β-pyrrolic and up to 2 axial positions) allow a wide variety of chemical functionalizations, so that a fine tuning of the electronic and photo-physical properties can be achieved quite easily.

Starting from 2010 up to now the solar energy to power conversion efficiencies of DSSCs based on porphyrins have increased remarkably, reaching values higher than those achieved with the best Ru^II^ dyes with the Zn^II^ complex coded YD2-oC8 co-sensitized with Y123 organic dye (12.3%) (Yella et al., 2011) and even higher (about 13%) with porphyrins SM315 (Mathew et al., 2014) and GY50 (Yella et al., 2014). Therefore, the expression “Porphyrin-Sensitized Solar Cells” has appeared in the literature (Li and Diau, 2013).

Nevertheless, porphyrin dyes still show some issues that must be addressed, such as the lack of absorption between the B and the Q bands and weak absorption in the NIR range of wavelengths.

Moreover, while *trans*-A_2_BC architectures have been extensively studied, including a very recently review (Lu et al., 2018), A_4_ β-pyrrolic substituted ones have received less attention, despite the simpler synthetic route and the more hindered structure, appealing for DSSCs.

Indeed, the synthesis of *trans*-A_2_BC Zn^II^ porphyrins involves the preparation of highly unstable unsubstituted dipyrromethane by reaction of pyrrole and formaldehyde, followed by a condensation step of dipyrromethane with a suitable arylaldehyde to afford the starting *trans*-A_2_ type porphyrin core, with a 25–30% overall yield. The subsequent bromination of the two free *meso* positions of the core affords a building block for further asymmetric functionalization with donor and acceptor moieties to produce a push-pull system. The different reactivity of the donor and acceptor pendant requires their stepwise introduction, thus lowering the yields and making the synthetic procedure rather ineffective (Yella et al., 2011; Mathew et al., 2014).

On the other hand, the synthesis of A_4_ β-pyrrolic architectures is less demanding. The core can be easily obtained by a one-pot condensation between pyrrole and the appropriate arylaldehyde with yields in the range 10–50% depending on the steric hindrance of this latter (Di Carlo et al., 2013, 2015; Li and Diau, 2013; Orbelli Biroli et al., 2015). After mono-bromination of the core in β-pyrrolic position and complexation with Zn^II^ (Di Carlo et al., 2015), a microwave-assisted Sonogashira coupling allows the direct introduction of the ethynyl pendant carrying the carboxylic or the cyanoacrylic acceptor and anchoring group with a 2-fold yield with respect to the thermal reaction (Di Carlo et al., 2013).

For a more detailed discussion on the synthetic pathways to *trans*-A_2_BC and A_4_ β-pyrrolic Zn^II^ porphyrins the reader is addressed to other reviews (Di Carlo et al., 2018 and references therein).

This mini-review aims to focus on the most common strategies reported so far in the literature to improve the light harvesting of these latter class of porphyrin-based dyes. In particular, we will highlight the relation among the molecular structure, the electronic properties and the spectral response, showing how the energy levels of the dye can be properly tuned through an *ad-hoc* molecular engineering and comparing A_4_ β-substituted structures with the analog *trans*-A_2_BC.

We will also discuss the methodologies to attain panchromatic sunlight capture covering the aspects related to the use of additional chromophores as co-adsorbents or as ancillary ligands tethered to the porphyrin molecular structure, including coordination of *ad-hoc* ligands to the metal center in axial position.

Other strategies including conjugation of two or more porphyrin molecules and the use of porphyrin cores fused together will be also reviewed as viable ways to improve spectral response up to the NIR region. Advantages and disadvantages of the surveyed approaches will be summarized trying to show the upcoming challenges to boost the light collection.

The reader is addressed to several comprehensive reviews (Hagfeldt et al., 2010; Clifford et al., 2011; Li and Diau, 2013; Gong et al., 2017; Song et al., 2018) for the description of the approaches that has been exploited for the optimization of the other key variables affecting the performances of a DSSC (electrolyte redox couple, dynamics of charge transfer processes, absorption of the dye on the TiO_2_ surface).

## Energy Levels and Spectral Response in Porphyrins and Metalloporphyrins

Porphyrins is a general term used to indicate a wide class of 18 π electron conjugated macrocycles, which are based on a specific molecular skeleton made up of four pyrrole rings, interconnected at their α carbons via methine bridges, and variously functionalized.

**Figure 2:**
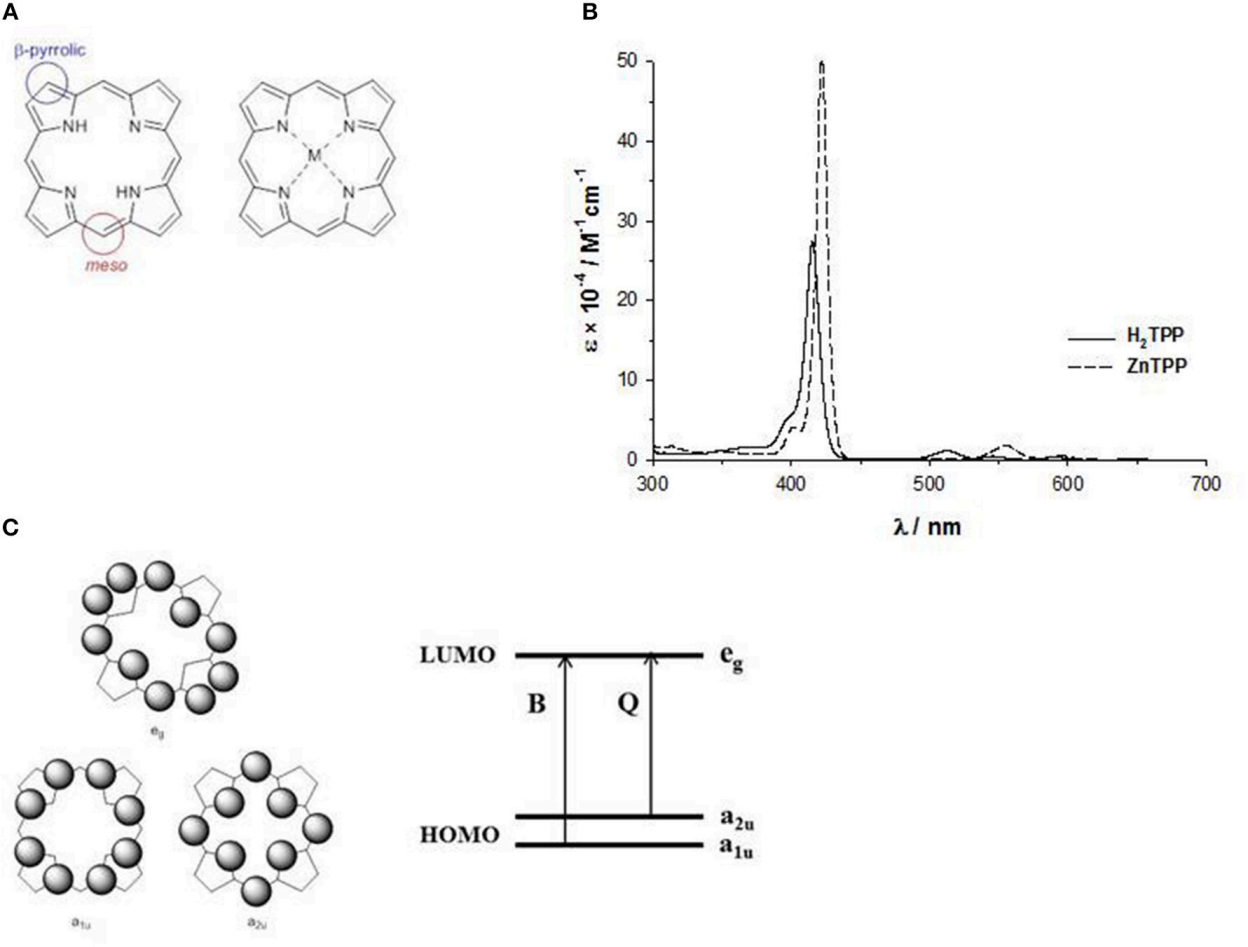
**(A)** *meso* and β-pyrrolic positions in porphine; **(B)** UV-Vis spectrum of H_2_-tetraphenylporphyrin and Zn^II^-tetraphenylporphyrin in THF solution; **(C)** HOMO and LUMO orbitals according to the four orbital model of Gouterman.

The basic structure, more correctly called porphine, is a planar ring with eight β-pyrrolic and four *meso* carbons suitable for chemical modifications, and the four nitrogen atoms outline an inner cavity able to bind metal ions, acting as a dianionic ligand (Figure 2A). Indeed, the two protons on the central nitrogen atoms are extremely weakly acidic (p*K*_a_ = 16).

All these compounds show intense colors ranging from red to green, depending on the chemical functionalization, the presence and the kind of metal ion and to a lesser extent, the solvent/environment. For example, in the biological field, the iron porphyrin devoted to oxygen transport and metabolism, called heme, is red colored, whilst the magnesium porphyrin at the base of the light harvesting process of the photosynthesis, chlorophyll, is yellow-green, but it gives to plants different green colors depending on the different protein environment, specific for each species.

Actually, the color of the simpler synthetic structures of these molecules is purple, and the name porphyrin is strictly connected color since it takes place from the place from the ancient Greek *porphura* (π*o*$\rho \phi \overset{´}{\upsilon }\rho \alpha$) used for purple pigments.

Color is only the fraction of visible light reflected, and it is complementary to the visible regions of the spectrum where light absorbance occurs. Therefore, UV-Vis absorption spectroscopy is a fundamental tool to study the properties of light harvesting and accordingly the energy levels of a dye. The knowledge of the dye spectroscopic features and the identification of their relation to molecular structure is of primary importance to plan tailored modifications for specific purposes.

The absorption spectrum of porphyrins show a typical and well-distinguishable pattern consisting in an intense band in the near UV region (380–450 nm) called B- or Soret band with a molar extinction coefficient in the order of 10^5^ M^−1^cm^−1^, and a series of weaker bands in the range 500–700 nm called Q-bands, with molar extinction coefficients of 10^4^ M^−1^cm^−1^.

The absorption spectrum of porphyrins covers a wide range of wavelengths of the solar emission and in particular the absorbance maximum of the B-band roughly corresponds to the maximum intensity of the solar spectrum: this is the reason why Nature selected this kind of molecules as light-harvester antennae at the base of the photosynthesis in plants.

However, also the role of the Q-bands is important, since they have a well-defined charge-transfer character (De Angelis et al., 2007) and their shift in the absorption range can change the resulting color.

Free-base porphyrins show four Q-bands (labeled I, II, III and IV from the lower to the higher energy band) with different relative intensity depending on the modification at the β-pyrrolic and *meso* positions of the macrocycle. In the simplest case, when the β-pyrrolic positions are not endowed with conjugated substituents, the intensities are IV>III>II>I and porphyrins are classified as *etio-type*. Other relative configurations of intensities ratio are possible: II>IV>II>I for *rhodo-type* featured by a red shifted spectrum and III>II>IV>I for *oxo-rhodo-type* with a more pronounced red shift spectrum. In these latter cases, the substituents in β-position have π-electrons able to interact with the π-structure of the porphyrin, and in particular for the *oxo-rhodo-type* these substituents are present at the opposite pyrrole rings. When also the *meso*-position is involved in the β-substitution, the relative intensities changes in IV>II>III>I and porphyrins are indicated as *phyllo-type*.

The inner cavity of the free-base porphyrin can react with protons leading to dication species, or with metal ions leading to metalloporphyrins. In both cases, the number of the Q-bands changes from four to two bands, suggesting a change of symmetry (Figure 2B). In fact, the free base porphyrin has a rectangular shape with a *D*_2h_ symmetry, but with the addition of protons or metal ions, produces a more symmetrical ring with a *D*_4h_ symmetry. For example, in Figure 2B, we report the UV-Vis spectra in THF solution of a free base porphyrin and of the corresponding Zn^II^ complex.

The two Q-bands of metalloporphyrins are labeled as α (the one at higher energy) and β and their relative intensities are related to the kind of metal and its type of coordination. For example, when the metal forms a stable square-planar complex, the α Q-band intensity is higher than that of the β Q-band, whereas when the metal complex is weak and protons can easily replace the metal, the intensities of the two Q-bands are reversed (Giovannetti, 2014).

Depending on the metal center, metalloporphyrins have been divided in two broad classes.

In *regular* metalloporphyrins, the closed shell metal cation (e.g., Mg^II^, Al^III^, Zn^II^, Cd^II^) produces only a small perturbation on the π-system of the porphyrinic ring. Therefore, the absorbance spectra are generally not influenced by the presence of the metal: only slight wavelength shifts to red of the B- and Q-bands are observed (Gouterman, 1978).

*Irregular* metalloporphyrins contain metal ions with partially filled shells, therefore a stronger mixing with the ring orbitals occur producing *hypso* or *hyper* absorption spectra (Gouterman, 1978).

*Hypso* absorption spectra are featured by a blue shifted pattern, and occur when the metal configuration is *d*^*m*^ with *m* = 6–9 (e.g., Fe^II^, Co^III/II^, Ni^II^, Cu^II^, platinum group metals). Instead *hyper* absorption spectra are featured by additional bands in the region above 320 nm, and occur in the presence of lower excited states metals (e.g., Sn^II^, Pb^II^) and or with transition metals with *d*^*m*^, 1 ≤ *m* ≤ 6 configuration (e.g., Cr^III^, Mn^III^, Os^VI^) (Gouterman, 1978).

The pattern of the absorption spectra of porphyrins has been clarified by the introduction of the so-called “four orbital model” proposed by M. Gouterman at the beginning of the ‘60s. According to this model, the B and the Q bands depend on the transition of two HOMOs and two LUMOs energy levels calculated by the Hückel theory. The HOMOs are two accidentally degenerate orbitals with a_1u_(π) and a_2u_(π) symmetry and an electronic density mainly located on the *meso*-positions and on the nitrogen atoms of the ring, whilst the LUMOs are a double degenerate orbital with e_g_(π^*^) symmetry and an electron density on the β-pyrrolic and *meso*-positions (Figure 2C) (Gouterman, 1961). The electronic configuration of the ground state is (a_1u_)^2^(a_2u_)^2^, yielding a singlet ground state (S_0_). The lowest excited state configurations are (a_1u_)^2^(a_2u_)^1^(e_g_)^1^ and (a_1u_)^1^(a_2u_)^2^(e_g_)^1^, giving rise to singlet and triplet excited states.

The electronic transitions between the HOMO and LUMO orbitals produce two excited states. The B or Soret band, with higher energy state and higher oscillator strength, is strongly allowed and can be indicated as the S_0_ → S_2_ transition (from the ground state to the second excited state), whereas the Q bands, with a lower energy and oscillator strength, are weakly allowed and can be indicated as the S_0_ → S_1_ transition (from the ground state to the first excited state).

The UV-Vis spectrum of porphyrins with 2 well-distinct absorption bands in different regions and characterized by S_0_ → S_2_ and S_0_ → S_1_ transitions, recalls the spectra of acenes, organic compounds made up of linearly fused benzene rings. Like acenes, porphyrins present fluorescence emission spectra from S_1_ → S_0_ transition according to the Kasha's rule. The emission spectrum is usually the mirror image of the of the Q-bands and can be obtain by exciting the sample both in the Q-bands region than in the B band region, since also the S_0_ → S_2_ transition contributes to populate the S_1_ excited state by non-radiative processes. In addition, the distance between the lower energy maximum peak in absorbance and higher energy peak defines the Stokes shift, which is an important indication of different configuration of the molecule between the ground and the excited states: the narrower is this shift, the more rigid is the molecule.

Recording the emission spectrum of a porphyrin is also of fundamental importance since its interception with the absorption spectrum allows the evaluation of the E^0−0^ energy, which corresponds to the spectroscopic HOMO-LUMO energy gap.

The HOMO-LUMO energy gap can be also inferred by DFT and TDDFT calculations of the energies and electronic distributions of the ground- and excited-state levels, using software packages such as Gaussian 03 (Frisch et al., 2004) or 09 (Frisch et al., 2009) and optimizing the structures *in vacuo* by the B3LYP functional (Becke, 1993) and basis sets such as 6-311G^*^ (Curtiss et al., 1995). When necessary, solvent effects are included by the C-PCM conductor-like solvation model (Cossi et al., 2003).

Finally, the HOMO-LUMO energy gap can be derived also by electrochemical measurements (Kadish and Van Caemelbecke, 2003), which provide information on the redox properties of the dye, through the experimental determination of the ground- and excited-state oxidation potentials, allowing an assessment of the charge injection and dye regeneration processes in the DSSC.

The electrochemistry of porphyrins usually presents two oxidation and two reduction processes, where stepwise one electron and a second one are removed or added to π system of the molecule respectively. The HOMO energy level corresponds to the first oxidation process and the LUMO to the first reduction process observed. Usually, all the peaks are reversible or quasi-reversible from both the electrochemical and the chemical point of view, allowing the calculation of the formal potentials E^0^′_Ia_ and E^0^′_Ic_ for the first oxidation (anodic) and first reduction (cathodic) processes, respectively, in the operating solvent (formal potentials E^0^′ approximate standard potentials E^0^ under the assumption of neglecting activity coefficients). From the experimental E^0^′ values the electrochemical HOMO and LUMO energy levels and the electrochemical HOMO–LUMO energy gap can be evaluated (Mussini et al., 2012), employing the ferrocenium/ferrocene redox couple as a reference for intersolvental comparison of electrode potentials (Gritzner and Köta, 1984; Gritzner, 1990) (Equations 4, 5).

(4) $$\begin{array}{r} E_{LUMO}\left(eV\right)=-e\times \left[\left(E_{Ic}^{0^{{\;}^{\prime }}}/V\left(Fc^{+}|Fc\right)+4.8\;V\left(Fc^{+}|Fc\;vs\;zero\right)\right)\right] \end{array}$$

(5) $$\begin{array}{r} E_{HOMO}\left(eV\right)=-e\times \left[\left(E_{Ia}^{0^{{\;}^{\prime }}}/V\left(Fc^{+}|Fc\right)+4.8\;V\left(Fc^{+}|Fc\;vs\;zero\right)\right)\right] \end{array}$$

However, electrochemical LUMO is different, in principle, from the optical LUMO, in fact the first reduction process leads to the formation of a radical anion species, whereas in absorption experiments, when an electron is promoted from the HOMO to populate the LUMO, the molecule results neutral from the electronic point of view.

On the contrary, the electrochemical HOMO well-describes the energy level of the radical cation species formed after the electron injection in the DSSC.

Therefore, for a correct evaluation of the LUMO level equation 6 can be used:

(6) $$\begin{array}{r} E_{LUMO}=E_{HOMO}-E^{0-0} \end{array}$$

## A_4_ β-Pyrrolic Substituted Zn^II^ Porphyrins as Light Harvesters

Although the electronic absorption spectrum of porphyrins and metalloporphyrins covers a wide range of wavelengths of the solar emission, the weak absorptivity between the Soret and the Q bands and the lack of absorption in the NIR are a severe limitation to their widespread use in DSSCs.

Typically, to address the lack of absorption between the Soret and the Q bands and to get a panchromatic spectral response, tremendous effort has been made, up to now, on the fine tailoring of the molecular structure of porphyrin dyes or on the use of complementary light harvesters.

The additional chromophore, showing a light-absorption pattern complementary to that of the sensitizers, can be directly linked to the porphyrin molecular structure or it can be used as a co-sensitizer of the semiconductor surface.

Although the co-adsorption of multiple dyes is considered a powerful method to boost the photon-collection of a DSSC, this approach strongly suffers from limitation about the choice and the loading optimization of selected dyes. Therefore, for this particular topic, we address the reader to other comprehensive reviews (Higashino and Imahori, 2015; Song et al., 2018).

In general, to be suitable as a dye for DSSCs, a molecule must have a LUMO level at higher energy than the CB of the semiconductor, to guarantee enough driving force for the electron injection, and a HOMO level at lower energy than the redox couple, for an efficient regeneration.

Focusing on the molecular engineering of porphyrins, their spectral response can be tuned by the introduction of suitable donor and acceptor moieties in the core, which affect the energy of the HOMO and LUMO levels, respectively, and therefore the electronic properties, the absorption spectrum and the light harvesting properties (Walsh et al., 2006; Mussini et al., 2012).

**Figure 3:**
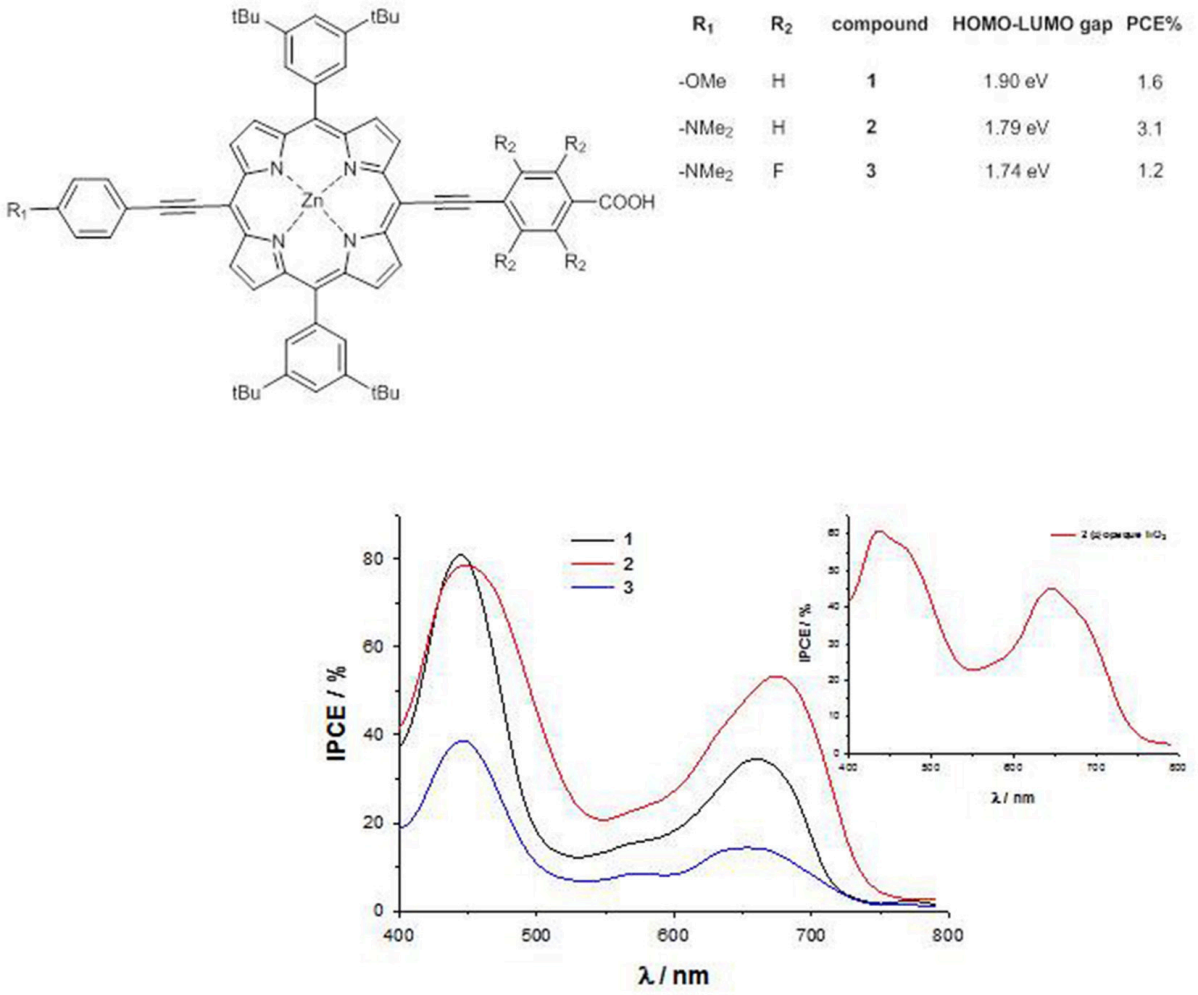
*trans*-A_2_BC push-pull Zn^II^ porphyrins **1-3** and their IPCE spectra.

For a series of *trans*-A_2_BC push–pull Zn^II^ porphyrins (Figure 3), a sizable destabilization of the HOMO energy level occurs by substitution of the weak electron-donor methoxy group with the stronger dimethylamino group (E_HOMO_ = −5.19 eV for **1**, −5.00 eV for **2** and −5.06 eV for **3**), together with a lowering in energy of the LUMO level by introduction of four fluorine atoms in the acceptor part of the molecule (E_LUMO_ = −3.29 eV for **1**, −3.21 eV for **2** and −3.32 eV for **3**) (Orbelli Biroli et al., 2011; Mussini et al., 2012). Therefore, the computed HOMO-LUMO energy gap decreases on going from dye **1** to **3**, pointing out for this latter a potential better charge-transfer process along the push-pull axis.

The cyclic voltammetry of compound **1** shows a peak at 0.91 V vs. SCE, due to the oxidation of the macrocycle core, while in **2** a slightly lower oxidation potential (0.87 V vs. SCE) is recorded, accompanied by a second wave at 0.72 V vs. SCE, due to the oxidation of the dimethylamino group (Mussini et al., 2012). In **3**, this latter oxidation is strongly anodically shifted, becoming a shoulder of a broad wave at 0.98 V vs. SCE. The calculation of the first-excited state potential by Equation (5) leads to values with the same trend than the calculated LUMO energies (−1.03 V vs. SCE for **2** > −0.92 V vs. SCE for **1** > −0.80 V vs. SCE for **3**).

The IPCE of **1**-**3** absorbed on TiO_2_ show 2 well-separated peaks, one at about 450 nm, corresponding to the Soret band, and the other at about 650 nm, corresponding to the Q band, with an intensity which decreases in the order **2** > **1** >> **3**.

Therefore, despite the lower HOMO-LUMO gap within the series, the very low IPCE values of dye **3** (40% at 450 nm and 20% at 650 nm) lead to poor photocurrents (*J*_*sc*_ = 4.5 mAcm^−2^) and photovoltaic performances (PCE = 1.2%), due to the lower charge injection efficiency and the increased charge recombination (Orbelli Biroli et al., 2011).

**Figure 4:**
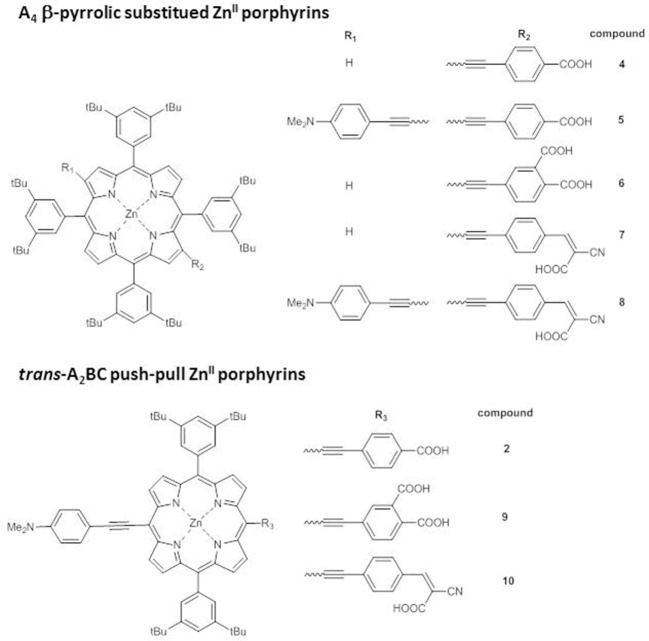
A_4_ β-pyrrolic Zn^II^ porphyrins **4-8** and *trans*-A_2_BC push-pull Zn^II^ porphyrins **9-10**.

Endowing the porphyrin with donor/acceptor moieties can lead to different effects also according to the structural architecture of the core, as pointed out by an investigation on *trans*-A_2_BC push-pull Zn^II^ porphyrins and on the analogs A_4_ β-pyrrolic structures (Figure 4) (Di Carlo et al., 2013).

**Figure 5:**
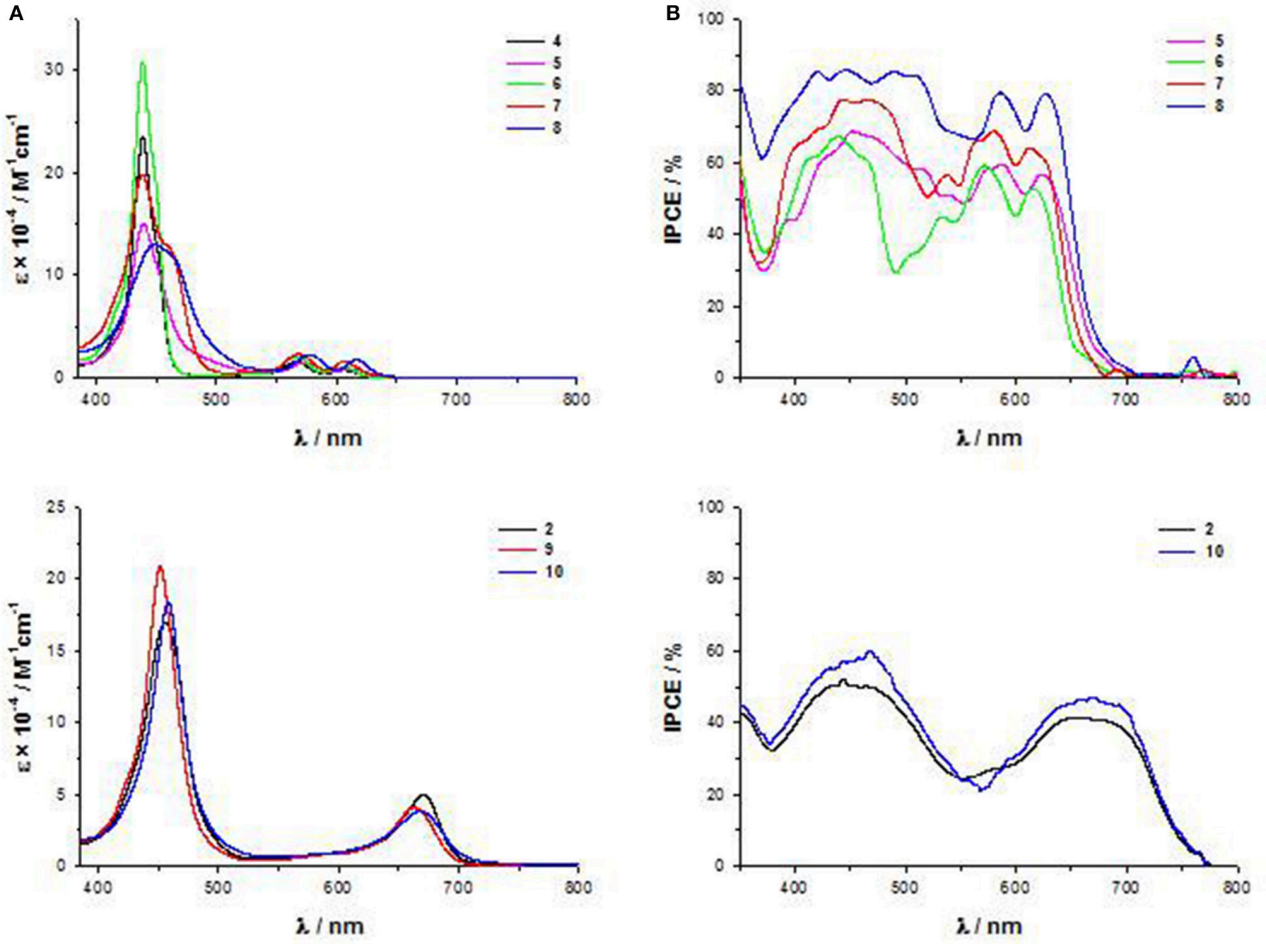
**(A)** UV-Vis spectra in THF solution of **4-10**; **(B)** IPCE spectra of **5-8** and **2** and **10**.

First of all, while the electronic absorption spectra of β-pyrrolic mono and di-substituted Zn^II^ porphyrins **4**-**8** show the typical pattern of metalloporphyrins with an intense B band in the range 430–460 nm and two weaker Q bands at 560–580 and 600–620 nm, *trans*-A_2_BC structures **2**, **9**, and **10** display in addition to the B band only one Q band, significantly stronger and red-shifted in comparison to the two Q bands of the β-pyrrolic counterparts (Figure 5A).

Accordingly, the computed HOMO-LUMO energy gaps of **2**, **9** and **10** (1.84–2.02 eV) are lower than those of **4-8** (2.06–2.59 eV) (Table S1).

In particular, a comparison of the HOMO-LUMO gaps of di-substituted complexes **5-2** and **8-10** with the same donor and acceptor substituents shows a more efficient transmission of the push-pull electronic effect between donor and acceptor groups in 5,15 *meso* positions of the macrocycle than in 2,12 β-pyrrolic positions, in accordance with an investigation of the second order NLO properties of these kind of chromophores (Orbelli Biroli et al., 2017).

However, the PCEs of DSSCs based on di-substituted A_4_ β-pyrrolic compounds **5** and **8** are higher than those of DSSCs based on the corresponding *trans*-A_2_BC compounds **2** and **10**, due to more intense IPCE spectra for A_4_ β-pyrrolic compounds, partially covering the lack of absorption around 500–550 nm, whereas the spectra of **2** and **10** show, as previously described, 2 well-separated maxima (Figure 5B).

When the porphyrin is mono-substituted in β-pyrrolic position with an electron-withdrawing cyanoacrilic group (compound **7**), the spectrum displays a plateau in the range 400–550 nm, reaching a remarkable 70% IPCE value.

The effect of the presence of a cyanoacrylic anchoring group linked by an ethynylphenyl moiety to the β-pyrrolic position of a porphyrin ring can be understood considering the perturbation induced by this strong electron-withdrawing substituent to the Gouterman's four orbital model. The degeneracy of the LUMO and LUMO+1 orbitals is broken, with a remarkable stabilization of the LUMO energy level. Moreover, the LUMO+2 level becomes closer in energy to the LUMO+1, giving rise to a nearly degenerate LUMO+1 and LUMO+2 system (Di Carlo et al., 2013). As a result, the HOMO-LUMO energy gap decreases, and the electron density on the ethynylphenyl moiety linking the core to the anchoring group increases.

Indeed, in the UV-Vis spectrum of **7** a red-shifted shoulder of the B band appears (Figure 5A) (Di Carlo et al., 2013).

The addition of a strong electron-donating dimethylamino group linked to the core by an ethynylphenyl fragment (compound **8**) causes a bathochromic shift of all the absorption maxima in the electronic absorption spectrum (Figure 5A), with a further improvement of the IPCE value (80%) (Figure 5B).

DFT calculations have shown that the energy of the LUMO of **8** is similar to that of **7** (−3.09 and −3.08 eV, respectively, Table S1), while a strong destabilization of the HOMO energy level occurs (−5.42 and −5.15 eV, respectively, Table S1), due to the introduction of the dimethylamino group, with a significant decrease of the HOMO-LUMO energy gap.

In mono and di-substituted β-pyrrolic Zn^II^ porphyrins, and in *trans*-A_2_BC Zn^II^ porphyrins, the cyanoacrilic group is more effective than the carboxylic group in perturbating the energy of the LUMO level and therefore in lowering the HOMO-LUMO energy gap, leading to an enhancement of the intensity of the IPCE spectra and of the photovoltaic performances.

The electrochemical data (Table S1) show that in the presence of a dimethylamino group in both 2, 12 β-pyrrolic (compounds **5** and **8**) and *meso*-position (compounds **2**, **9**, and **10**) the first anodic peak shifts at lower potential, being located on the dimethylamino group, while the second oxidation involves the porphyrin core. Moreover, di-substituted β-pyrrolic Zn^II^ porphyrins **5** and **8** display more positive anodic peaks and more negative cathodic peaks than their *trans*-A_2_BC counterparts **2**, **9**, and **10**, confirming a wider HOMO-LUMO energy gap and a less efficient charge transfer for β-substituted porphyrins.

The electrochemical HOMO-LUMO gap of mono-substituted β-pyrrolic Zn^II^ porphyrins **4**, **6**, and **7** is higher as a result of the concomitant positive shift of the first anodic peak and a negative shift of the first reduction peak. However, in accordance to the DFT investigation, the introduction of a cyanoacrylic acceptor unit in **7** instead of a carboxylic one lowers both the oxidation and the reduction potentials, leading to lowest HOMO-LUMO gap in the mono-substituted series.

The most attractive approach to broaden light absorption is to link ancillary light absorbing substituents to the periphery of a porphyrin dye by an efficient π-conjugated system. This strategy has been widely used to improve the light harvesting ability of a number of porphyrin-based dyes. The elongation of π-conjugated acceptor systems on porphyrin molecules plays an important role in improving the photon collection ability of the dye, both in *meso* and β-substituents, giving rise to a better light harvesting over the whole visible spectrum and resulting in a panchromatic effect on the IPCE spectra and in an enhanced photocurrent, thus increasing DSSC performances (Di Carlo et al., 2014b; Arrechea et al., 2016).

In particular, the ability of dithienylethylene (DTE) to absorb within the 500–550 nm spectral region, where the absorption of more common porphyrins show poor harvesting and conversion has been exploited.

**Figure 6:**
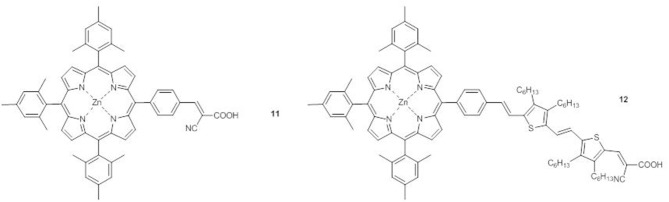
Zn^II^ porphyrins **11** and **12**.

When a DTE unit is added to compound **11** at the *meso*-position to give compound **12** (Figure 6), in the electronic absorption spectrum a new absorption between 430 and 630 nm appears, in addition to the B band and the two Q bands, due to the π-conjugated system of the DTE (Barea et al., 2011).

DFT calculations show a remarkable decrease of the HOMO-LUMO gap of **12** in comparison to **11** (2.18 and 2.62 eV, respectively), thanks to enhanced charge transfer and charge separation processes.

Therefore, the DSSC employing **12** as the sensitizer shows a triplication of the photocurrent and of the PCE (*J*_*sc*_ = 15.6 mAcm^−2^ and PCE = 4.77%) with respect to that with compound **11** (*J*_*sc*_ = 3.6 mAcm^−2^ and PCE = 1.10%), with an enhancement of the IPCE value to 60% and a remarkable flattening between 400 and 650 nm (Barea et al., 2011).

The same panchromatic approach has been proven to be effective also to enhance the light harvesting of A_4_ β-pyrrolic Zn^II^ porphyrins.

**Figure 7:**
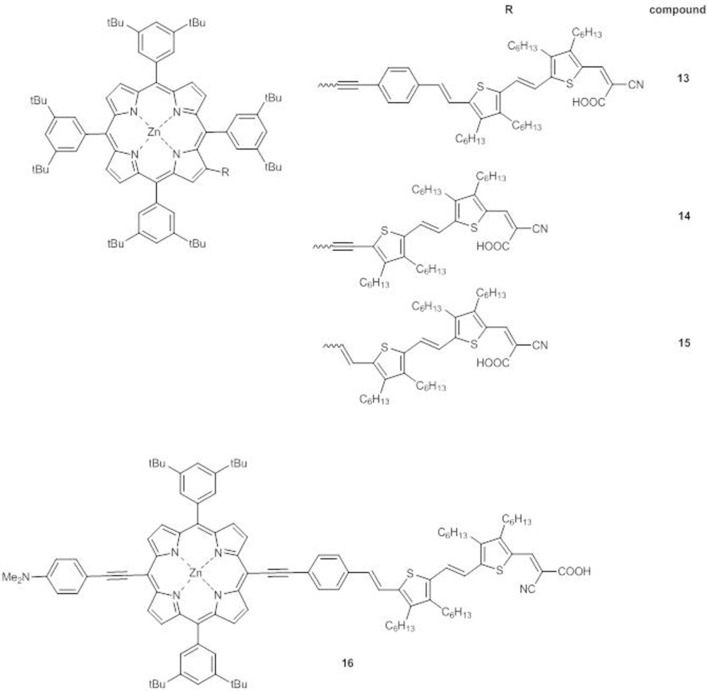
A_4_ β-pyrrolic Zn^II^ porphyrins **13-15** and *trans*-A_2_BC push-pull Zn^II^ porphyrin **16**.

In compounds **13-15**, a DTE moiety is linked to the core in β-pyrrolic position by different π-conjugated bridges (Figure 7) (Di Carlo et al., 2014a).

**Figure 8:**
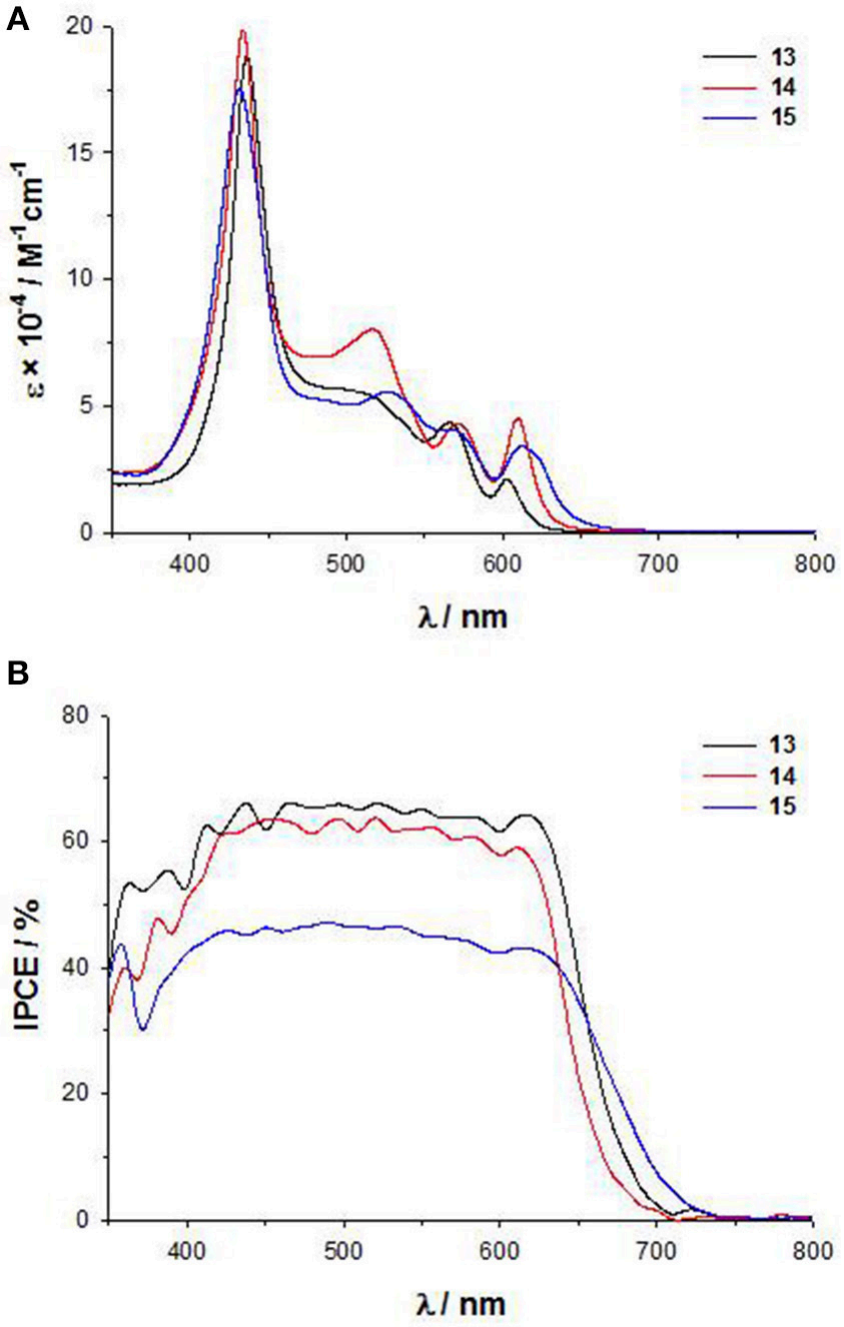
**(A)** UV-Vis spectra in THF solution of **13-15**; **(B)** IPCE spectra of **13-15**.

As for **11**-**12**, the introduction of a DTE moiety leads to the appearance in the UV-Vis electronic absorption spectra of **13**-**15** a new band between 490 and 530 nm, very intense for **14** (Figure 8A). In agreement, a broad plateau in the range 430–650 nm characterizes the IPCE spectra (Figure 8B), with comparable intensities for **13** and **14** (60%) and a lower value for **15** (40%). The electrochemical data (Table S1) support for all the three dyes HOMO and LUMO energies fully compatible with the requirements of a DSSC. However, in agreement with the IPCE spectra, **15** displays a lower photocurrent and a lower PCE than **13** and **14** (*J*_*sc*_ = 10.5 mAcm^−2^ and PCE = 4.3% for **15**, while for **13** *J*_*sc*_ = 12.4 mAcm^−2^ and PCE = 5.2% and for **14** *J*_*sc*_ = 11.7 mAcm^−2^ and PCE = 4.7%) (Di Carlo et al., 2014a).

Therefore, the performances of DSSCs based on these panchromatic dyes strongly depend on the bridge between the DTE unit and the porphyrinic core, which influences the structural arrangement and consequently, the charge separation involved in the electron injection process. Due to the ethynylstyryl and the ethynyl spacers, **13** and **14** have a more planar and linear spatial structure than **15**, which on the other hand appears hindered and distorted.

Transient optical studies at the femtosecond scale allowed to explore in-depth the impact of a DTE unit on the photophysical properties of a porphyrin dye (Di Carlo et al., 2017). A_4_ β-pyrrolic substituted Zn^II^ porphyrin **13** was compared to **7**, lacking the DTE unit, and to *trans*-A_2_BC Zn^II^ porphyrin **16** (Figure 7).

The photophysical investigation of charge transfer dynamics demonstrated that endowing a porphyrin core with a DTE unit has a remarkable effect in enhancing sunlight harvesting, regardless of the substitution in *meso* or β position, extending the spectral response of the dyes without introducing a kinetic barrier to charge transfer processes. Indeed, the comparative kinetic study shows ultrafast electron injection onto the photoanode at the femtosecond time-scale (*ca*. 200 fs), similar for all DTE endowed dyes (**13** and **16**, Figure 7) and the reference **7** (Figure 4) without the additional chromophore as well, suggesting other mechanisms involved in boosting the DSSC performances, more likely attributed to the extended spectral sensitization of panchromatic dyes. Besides, the injection rate of β-substituted porphyrins absorbed on TiO_2_ film was previously reported to be higher than N719 Ru-based dye (Sunahara et al., 2011) and higher or similar to the *meso*-substituted porphyrins (Campbell et al., 2004; Di Carlo et al., 2014b). A further time-resolved optical and electrical investigation focused on variously substituted porphyrinic dyes, revealed that the substituent position is not the determining factor of injection efficiency (Griffith et al., 2012). As a consequence, despite an increasing of the push-pull character clearly affects the spectral response by broadening and red shifting the absorption bands, the impact on the electron injection rate (subpicosecond time-scale) cannot rationally explain the differences in PV efficiency among structurally different porphyrin-based dyes. However, the charge recombination processes between sensitized TiO_2_ and the dye cation or the oxidized electrolyte, are typically on a longer time scale (ns-ms) and much more likely involved in influencing the DSSC performances.

Grätzel and co-workers in 2014 by a judicious molecular engineering of *trans*-A_2_BC push–pull Zn^II^ porphyrins dramatically improved the light harvesting ability of the SM315 dye reaching the record 13% conversion efficiency for porphyrin-based DSSC (Mathew et al., 2014).

The introduction of the proquinoidal benzothiadiazole (BTD) unit into the acceptor pendant of the dye promoted a splitting of the B band, resulting in a shoulder at 440 nm added to the maximum at 454 nm, with a significant broadening of the electronic absorption between the B and the Q bands (450–550 nm) and a bathochromic shift with increased intensity for the Q band at lowest-energy. Therefore, an 80% IPCE value with enhanced photon collection in both the green (500–600 nm) and red (up to 800 nm) region of the solar spectrum was achieved, resulting in a panchromatic capture of sunlight which allowed the DSSC device to get enhanced photocurrent and outstanding efficiency without the use of other co-sensitizers (Mathew et al., 2014).

Besides BTD, other auxiliary acceptor units based on 2,3-diphenylquinoxaline (DPQ), benzotriazole and triazolopyridine moieties have been recently investigated by bridging the core with carboxylic anchoring groups in *trans*-A_2_BC Zn^II^ porphyrins (Lu et al., 2016; Cheng et al., 2017).

The presence of these auxiliary groups promotes a panchromatic effect on the IPCE spectra in the range from 350 and 700 nm, filling up the valley between the B and the Q-bands and enhancing sunlight harvesting up to 60–70%.

A DPQ and a BTD moiety show similar effects also when added in β-pyrrolic position (Lu et al., 2017).

**Figure 9:**
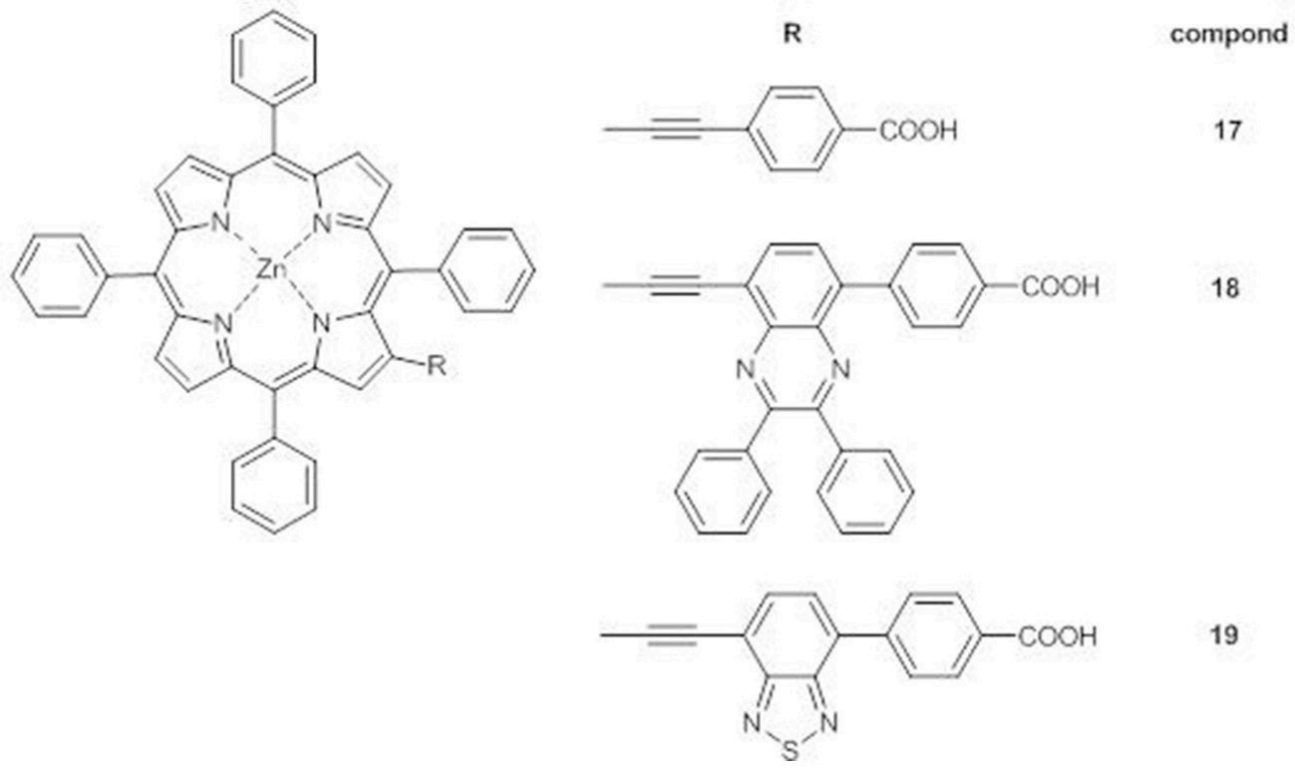
A_4_ β-pyrrolic Zn^II^ porphyrins **17-19**.

When compared to the reference dye **17**, compounds **18** and **19** (Figure 9) display a splitting and a red-shifting of the electronic absorption bands, due to the introduction of the additional electron-withdrawing group which enhances the π-conjugation and the electron transfer between the porphyrin core and the anchoring carboxylic group. Moreover, as for the *meso*-substituted analogs (Lu et al., 2016; Cheng et al., 2017), a shoulder of the B band appears (at 464 nm for **18** and at 471 nm for **19**), more bathochromically shifted for **19**, in accordance to the more electron-acceptor nature of BTD with respect to DPQ.

DFT calculations have shown that the HOMOs of all three **17**-**19** are mainly on the porphyrin core and have almost the same energy, while a stepwise stabilization of the LUMOs occurs on going from **17** to **19**, as expected for the increased electron-withdrawing properties of the additional acceptor unit and in agreement with the anodic shift of the first oxidation peak (Table S1).

Accordingly, the IPCE spectra of the DSSCs sensitized with **18** and **19** have higher and more extended absorptions in the range 350–520 nm than the one based on **17**, reaching an IPCE value over 60% for **19**. Indeed, **19** has the highest photocurrent (*J*_*sc*_ = 11.47 mAcm^−2^ vs. 7.60 mAcm^−2^ for **17** and 9.03 mAcm^−2^ for **18**) and the highest PCE within the series(6.14 vs. 4.02% for **17** and 4.47% for **18**).

Hence the introduction of supplementary electron-withdrawing moiety in the acceptor unit is a valid strategy to extend light harvesting ability of both *meso* and β-substituted porphyrins, with a more pronounced effect in combination with BTD-based acceptors. In fact, almost all the new generation porphyrin dyes carry this latter moiety as an ancillary acceptor between the porphyrin core and the anchoring group (Pan et al., 2018).

The use of an additional chromophore to improve sunlight harvesting has been exploited also for axially substituted porphyrins.

**Figure 10:**
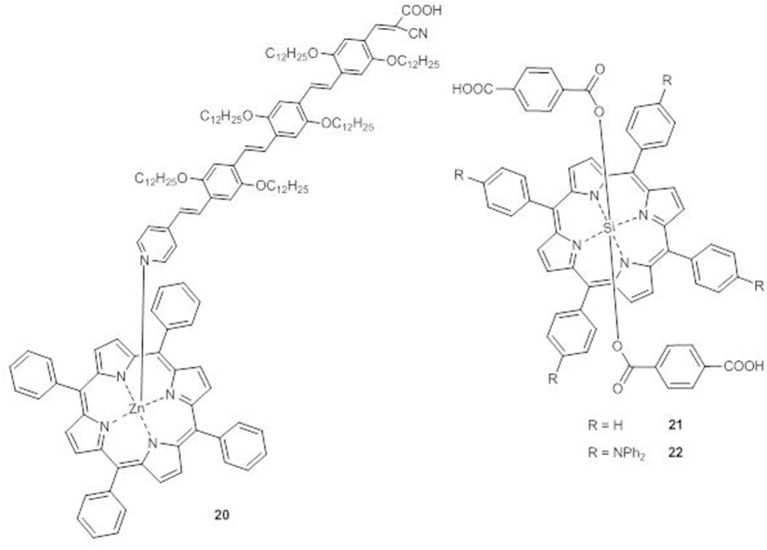
Axial porphyrins **20-22**.

A supramolecular dyad (**20**) between Zn^II^ tetraphenylporphyrin and a π-conjugated oligo(phenylenevinylene) moiety with a pyridyl group able to interact with the metal center and a cyanoacyrylic anchoring group to bind TiO_2_ has been prepared (Figure 10) (Charisiadis et al., 2015).

The DSSC sensitized with **20** shows an IPCE spectrum broader than that of the DSSC built with the plain oligo(phenylenevinylene) moiety, reaching IPCE values of 35 and 40% at 422 and 486 nm, respectively. Therefore, a significant enhancement of *J*_*sc*_ (from 7.55 to 11.58 mAcm^−2^) and of PCE (from 2.45 to 5.27%) occurs by axial coordination.

In porphyrins **21** and **22**, the central silicon atom allows the coordination of two terephthalic acid units as axial ligands and anchoring groups (Figure 10) (Liu et al., 2013).

The UV-Vis spectra of **22** in THF solution shows an absorption band at 411 nm in addition to the B and the Q bands, which are also significantly red-shifted in comparison to those of **21**, due to the presence of the triphenylamine units in the *meso*-positions of the porphyrin core.

In accordance to the enhanced electron-donating ability and to the increased conjugation of **22**, CV experiments with TiO_2_ photoanodes sensitized with the two dyes have shown a positive shift of the LUMO and a negative shift of the HOMO of **22** with respect to those of **21** (−0.84 V vs. NHE and −0.90 V vs. NHE, respectively, for the LUMOs and 0.96 vs. NHE and 1.00 V vs. NHE, respectively, for the HOMOs).

The DSSC with compound **22** displays a broader, flatter and more intense IPCE spectrum than **21**, with a tail reaching 750 nm and 40–60% peaks in the range 380–670 nm. The resulting photocurrent is more than twice that of **21** (*J*_*sc*_ = 8.24 vs. 3.28 mAcm^−2^) with a consistent improvement of the PCE (3.0% for **22** and 1.0% for **21**).

Since the first example of a porphyrin dye equipped with a push-pull system along the 5,15-*meso* positions in which the donor and the acceptor units are introduced in a *trans*-A_2_BC architecture (Lee et al., 2009), great efforts have been devoted on judiciously designing porphyrin sensitizers with such a kind of substitution pattern. This molecular architecture provides a significant charge transfer character from the donor to the acceptor through the porphyrin π-system guaranteeing an increased power conversion efficiency compared with analog dyes missing the donor substituents. The push-pull system definitely allows to boost the DSSC performance due to the increased photocurrent density on the semiconductor by pushing the electron flow from the dye to the TiO_2_ semiconductor.

The introduction in the push-pull framework of strong electron-donating substituents, such as diaryl-amine donors, not only positively affects the electron injection process, but it further improves the light harvesting ability of the sensitized photoanode by broadening and extending up to the NIR region the absorption bands of the porphyrin dye (Lee et al., 2009). Therefore, a large number of donors with increasing electron-donating strength have been designed and investigated to simultaneously enhance the conversion efficiencies and the photon absorption of the dyes by linking at the *meso*-positions of the core.

However, it is mandatory to keep in mind that too strong donors seriously destabilize the HOMO levels making the regeneration process of the dye by the electrolyte very hard to take place with a negative impact on the PCE (Figure 1).

**Figure 11:**
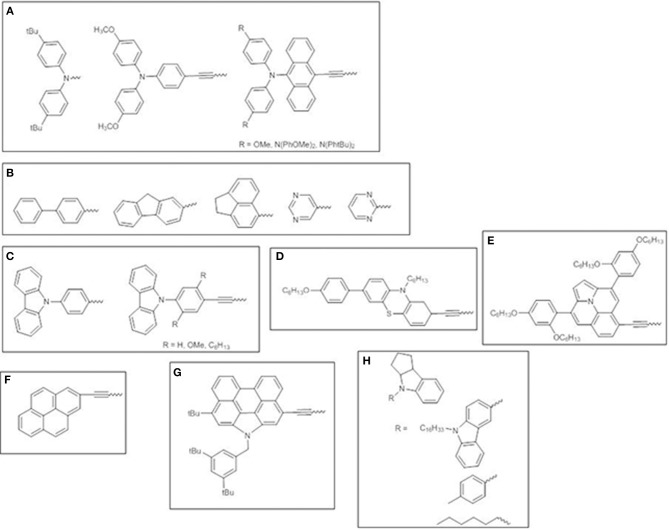
Various electron-donor moieties used for *trans*-A_2_BC push-pull Zn^II^ porphyrins. **(A)** arylamines; **(B)** polyaromatic or heterocyclic groups; **(C)** carbazole; **(D)** phenothiazine; **(E)** ullazine; **(F)** pyrene; **(G)** N-annulated perylene; **(H)** indoline-conjugates.

Besides aryl-amino donors, which have been extensively investigated (Figure 11A) (Lee et al., 2009; Hsieh et al., 2010; Chang et al., 2011; Pan et al., 2017), other electron-donating moieties have been surveyed. Fluorene-modified porphyrins have been reported to outperform other porphyrin dyes endowed with polyaromatic or heterocyclic donors, due to the more red-shifted absorption bands promoting stronger and broader photovoltaic responses in the range 350–750 nm (Figure 11B) (Wu et al., 2012).

Carbazole (Figure 11C) (Wang et al., 2013, 2014) and phenothiazine (Figure 11D) (Xie et al., 2015) donor moieties have also been investigated as promising donors for developing efficient porphyrin sensitizers. The introduction of an ethynyl bridge between the donor and the porphyrin core improved light absorption and expanded π-conjugated framework. The side effect for carbazole derivatives is the simultaneous increasing of dye aggregation, which has been overcome by incorporating long-alkoxy chains in the dye structure. On the contrary, the 3D shape of phenothiazine allowed the aggregation to be suppressed.

The introduction of an ullazine donor motif promoted significant absorptions in both the visible and NIR region (up to 800 nm) of the solar spectrum thus boosting the light harvesting ability and achieving a viable approach for designing panchromatic dyes (Figure 11E) (Mathew et al., 2016).

Some cyclic aromatic donors have been also investigated, such as pyrene (Wang et al., 2011) and N-annulated perylene (Luo et al., 2014) (Figures 11F–G), which show a remarkable panchromatic effect when linked to the porphyrin core by an ethynyl π-bridge, thus enhancing solar light harvesting in the range 400–800 nm and up to the NIR and producing IPCE values higher than 80% and superior PCEs, up to 10.5%.

Finally, indoline-conjugated units have been considered, producing DSSCs with improved photocollection over a wide range of wavelengths (350–700 nm, with IPCE values of 80%, surpassing those of benchmark dye YD2-oC8) and remarkable PCEs (Figure 11H) (Pellejà et al., 2014; Li et al., 2016). The indoline motifs show nonplanar geometry enabling to endow the dye molecules with a proper steric hindrance to reduce the molecular aggregation.

While several electron-donors have been exploited for the optimization of sunlight harvesting in *trans*-A_2_BC Zn^II^ push-pull porphyrins, a similar systematic investigation on A_4_ β-pyrrolic structures is still lacking, mostly because of some issues related to their preparation.

Complexes **5** and **8** have been the first examples of β-disubstituted push-pull Zn^II^ porphyrins ever reported (Di Carlo et al., 2013), thanks to the development of a straightforward and effective synthetic procedure involving a light induced regiospecific introduction of bromine atoms on antipodal 2,12 β-pyrrolic position of the porphyrin core (Di Carlo et al., 2015), followed by a microwave-assisted Sonogashira coupling.

As discussed before, it was evidenced that the porphyrinic ring is a less effective linker between the donor and acceptor substituents along pyrrolic positions. However, the introduction of the dimethylaniline donor in β-position gives broad and intense IPCE spectra in the range 350–650 nm, outperforming the PCEs of the corresponding *meso*-porphyrins. The pyrrolic substitution architecture enables to fill the valley around 500 nm typical of the *meso* series and the additional donor promotes red shifting of the Q bands compared with the β-substituted porphyrins bearing only the acceptor.

Following a similar approach, attractive A_4_ 2,12 β-pyrrolic disubstituted Zn^II^ porphyrins with a ferrocenyl moiety as a strong donor group and C60-fullerene or carbon spheres as acceptor units linked to the porphyrin core through an ethynyl bridge have been prepared (Possanza et al., 2018). The designed triads exhibit an appreciable broadening of the B band and a 20 nm bathochromic shift of both the B and the Q bands, making them promising candidates for optoelectronic and photovoltaic applications due to their increased photon-collection ability.

However, a more convenient strategy to enhance the push-pull character of A_4_ β-functionalized porphyrin dyes and tune their light-absorption profile is the introduction of electron-donating groups on the aryl moieties in 5,10,15,20 *meso* positions.

**Figure 12:**
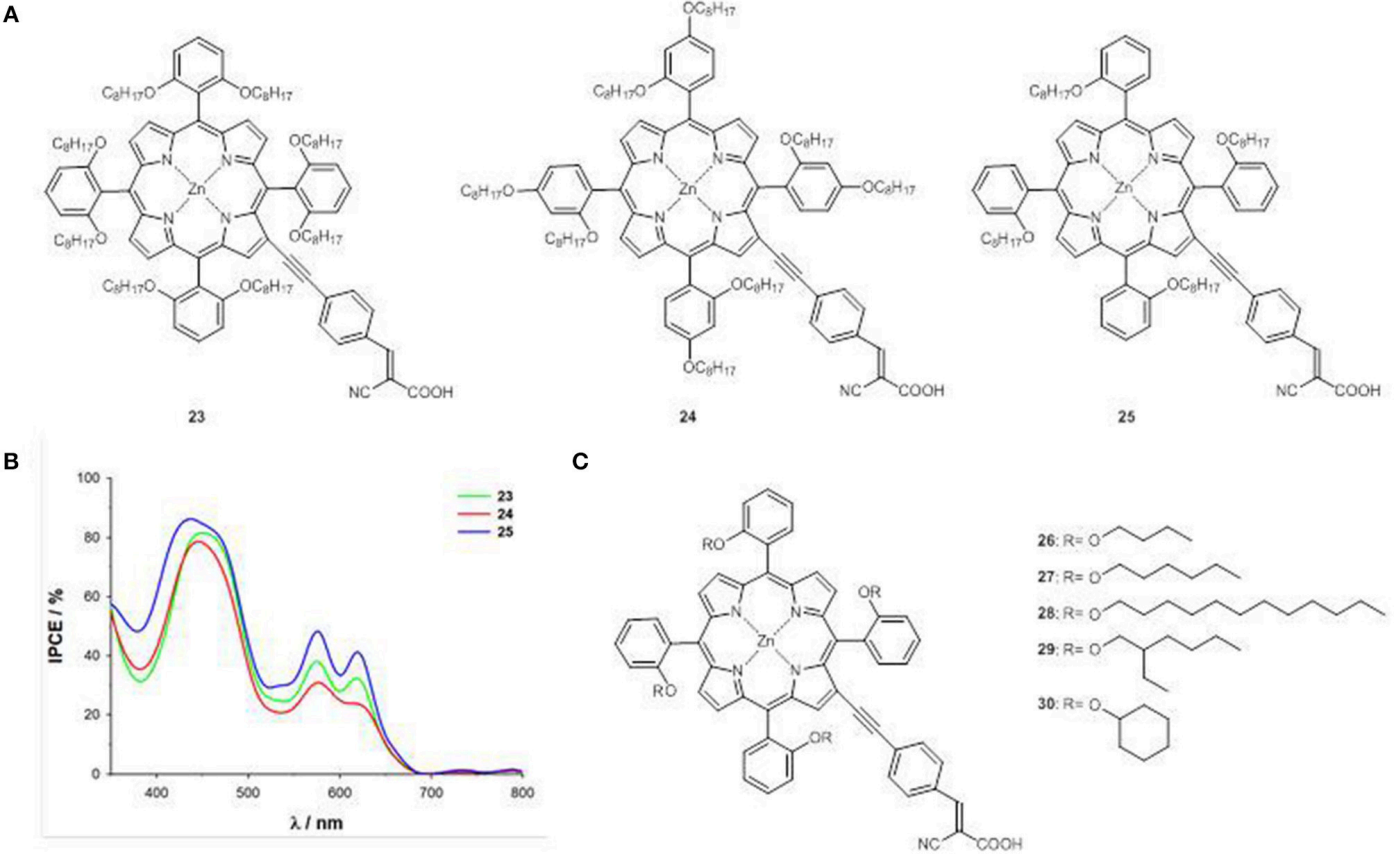
**(A)** A_4_ β-pyrrolic Zn^II^ porphyrins **23-25**; **(B)** IPCE spectra of **23-25**; **(C)** A_4_ β-pyrrolic Zn^II^ porphyrins **26-30**.

Following the approach used for the optimization of the photovoltaic performances of *trans*-A_2_BC push-pull Zn^II^ porphyrins by suppressing their detrimental π-π stacking (Li and Diau, 2013), the three new porphyrins **23**, **24**, and **25** have been prepared, carrying octyloxy chains at the *o,o*-, *o,p*- and *o*-positions of the phenyl moieties in *meso* of the macrocycle (Figure 12A) (Orbelli Biroli et al., 2015).

Compound **23** shows a surprisingly narrow electrochemical HOMO-LUMO energy gap (1.77 eV), very similar to that of *meso* push- pull Zn^II^ porphyrins **2**, **9**, and **10** in the same experimental conditions (Figure 4) (Mussini et al., 2012; Di Carlo et al., 2013), suggesting a remarkable role of the octyloxy chains in *o,o* position not only, as expected, on the reduction of detrimental aggregation phenomena, but also on the electronic structure and on the charge transfer processes.

The CV investigation has confirmed for **23** a low first oxidation potential (0.14 V vs. Fc^+^/Fc, Table S1), in agreement with an enrichment of the electronic cloud below and above the porphyrin plane due to the presence of eight oxygen atoms.

The PCEs of the DSSCs based on **23**-**25** show a significant increase compared to that with reference complex **2**, in particular for the device with **25** (80% increase), which displays the highest IPCE value within the series (Figure 12B).

Focusing on *o*-substituted structures, the effect of alkoxy chains of different length an steric hindrance has been considered (compounds **26**-**30**, Figure 12C) (Magnano et al., 2016).

The first anodic and cathodic peaks and therefore the electrochemical HOMO and LUMO energy levels and the HOMO-LUMO gaps, are almost unaffected by the change of the alkoxy chain (Table S1). On the contrary, the PCEs of the DSSCs made with **26**-**30** enhance linearly with the alkoxy chain length, due to an enhancement of both photocurrent and photovoltage, and reach the highest value (6.32%) for the device with **28**, in accordance with the IPCE spectra.

Longer alkoxy chains provide a better enveloping of the porphyrin core, reducing π-π aggregation and protecting the Zn^II^ ion by recombination with the ${\text{I}}_{3}^{-}$ of the electrolyte.

**Figure 13:**
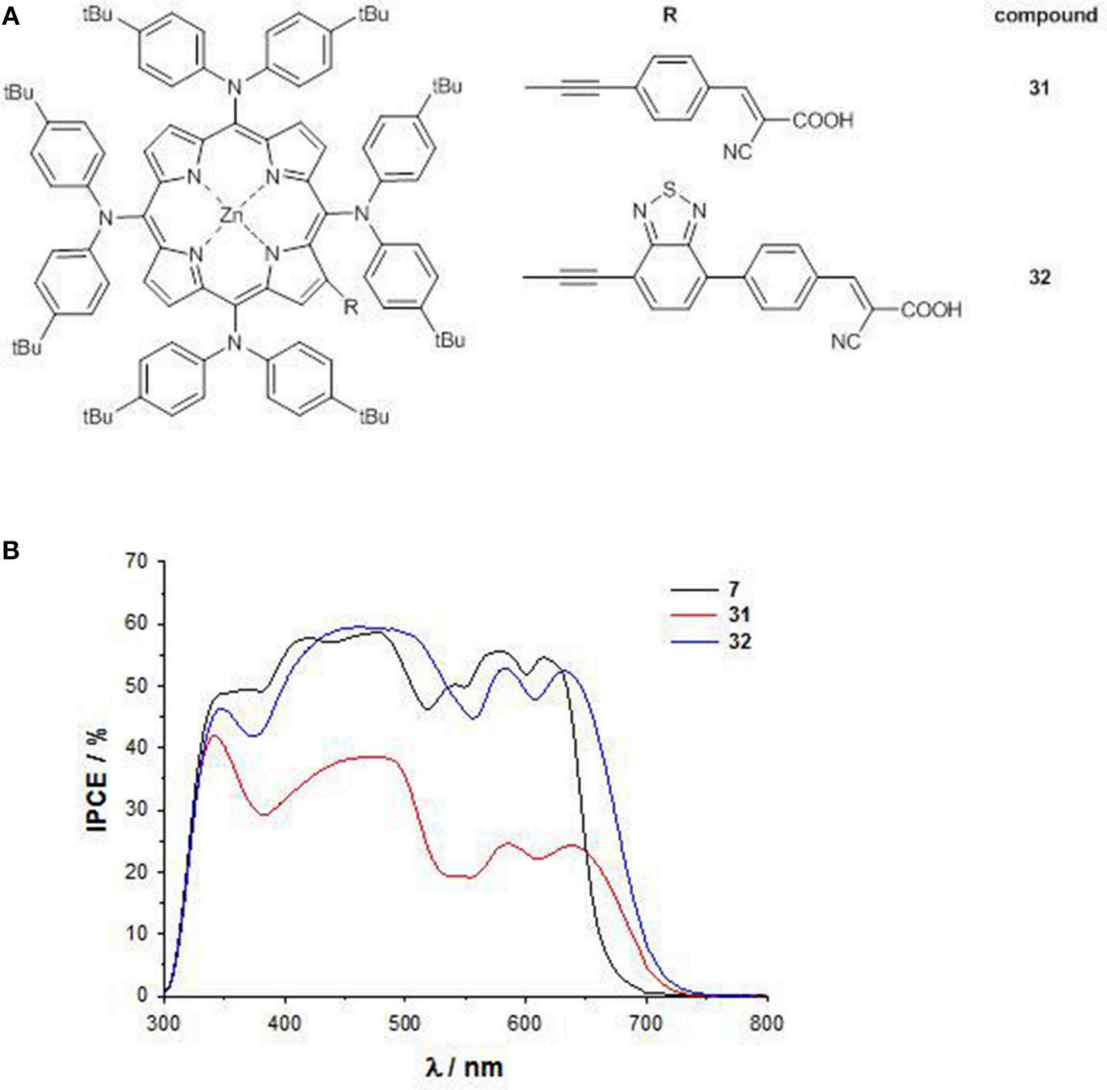
**(A)** 4D-π-1A porphyrins **31-32**; **(B)** IPCE spectra of **31-32**.

Pushing forward this approach, a novel 4D–π-1A type substitution pattern bearing four bulky electron-donating substituents directly linked to the 5,10,15,20 *meso* positions of the core and a β-pyrrolic electron-withdrawing pendant has been rationally designed (**31** and **32**, Figure 13A) (Covezzi et al., 2016).

Indeed, the conjugation of triphenylamine units on a porphyrin ring was previously reported to produce broadened photon absorption both in the visible and NIR regions in porphyrin dyes (Huang et al., 2012), so the introduction of these moieties was coupled with the simultaneous introduction of a BTD unit in the acceptor part of the molecule (compound **32**), to achieve a panchromatic response.

The significant destabilization induced on the HOMO energy levels by the four arylamino groups and on the LUMO energy levels by the BTD pendant (as reflected also by the less negative first cathodic potential, Table S1) allowed to lower the HOMO-LUMO gap of **32** in comparison to that of **31** (1.92 vs. 2.08 eV) and especially to that of reference compound **7** (2.33 eV), highlighting an enhanced push-pull character for porphyrins with 4D-π-1A substitution pattern.

Accordingly, the B and the Q band at lower energy of **31** and **32** are significantly red-shifted with respect to **7**.

The DSSC based on **32** shows an IPCE value up to 60% over a broad spectral range (300–700 nm), and a remarkable PCE of 8.79%. On the contrary, due to the shorter anchoring unit, the device with **31** suffers back electron transfer, showing therefore a lower (even if panchromatic) IPCE (<40%), and a small PCE (2.72%) (Figure 13B).

Therefore, sometimes the better light harvesting induced by a broader absorption profile fails in increasing the efficiency of the DSSC. Indeed, the peripheral substituents must be linked in a proper way to the porphyrin macrocycle in order to promote efficient overlapping of π-orbitals which guarantees adequate electronic communication among the chromophores composing the panchromatic dye (Obraztsov et al., 2017).

Typically conjugated ethynyl and ethenyl moieties are considered the most efficient linking bridges between the porphyrin core and the acceptor unit, while ethynyl and amine bridges are commonly preferred for the connection of donor moieties. The ethynyl bridge promotes extended harvesting of sunlight by broadening and red-shifting of the absorption bands (Di Carlo et al., 2014a), but, in the case of pseudo-planar structures, as *meso*-substituted porphyrins, such a linking way could dramatically aggravate the planarization of the dye, with a negative impact on the DSSC performances by π-staking aggregation of the dye molecules onto the semiconductor.

The introduction of alkyl chains on donor, acceptors or directly on the porphyrin ring was firstly considered to address these drawbacks (Orbelli Biroli et al., 2015; Magnano et al., 2016; Song et al., 2018).

Recently new substitutive patterns for β-pyrrolic substituted porphyrin dyes have been investigated.

**Figure 14:**
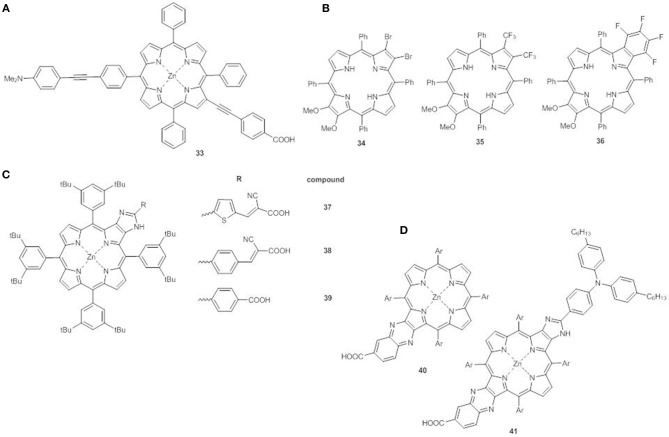
**(A)** *meso*-β disubstituted Zn^II^ porphyrin **33**; **(B)** multi β-substituted free base porphyrins **34**-**36**; **(C)** β-functionalized imidazole-fused porphyrin dyes **37-39**; **(D)** push-pull fused Zn^II^ porphyrins **40-41**.

For example, linking an electron donor group in *meso* position of the ring and an electron acceptor in β-pyrrolic position, both through an ethynyl spacer (compound **33**), generates an additional extended band at between the Soret and the Q-bands (500–600 nm) referred to as S_0_ → S_3_ excitation. This structural arrangement greatly enhances solar light harvesting in the green spectral region, making these new porphyrins promising candidates for DSSC application (Figure 14A) (Parsa et al., 2018).

β-multi-substituted free base porphyrins carrying electron-donor methoxy groups and various electron-withdrawing groups (Br, CF_3_, and C_6_F_4_) at the antipodal β-positions have also been explored (**34**-**36**, Figure 14B) (Chen et al., 2013). The distorted saddle configuration of these structures, coupled with the different electron-acceptor ability of the substituents, tunes the HOMO-LUMO energy gap, which decreases in the order **34**>**36**>**35** with the same trend for electrochemical and calculated data. The electronic absorption spectrum of **35** displays a broader B band than **34** and **36**, and stronger and red shifted Q bands (511 and 718 nm).

Another interesting design comprises the use of five-member hetero-aromatic rings condensed to the pyrrolic rings of the porphyrin as alternative linking bridge enabling efficient electron transfer without increasing the steric hindrance.

The β-functionalized imidazole-fused porphyrin dyes **37-39** (Figure 14C) display different HOMO-LUMO gaps and light harvesting properties which depend on the π-bridges and the acceptor groups on the fused imidazole-porphyrin donor (Bodedla et al., 2018). The trend of the first oxidation potentials (**37**<**38**<**39**, Table S1) is related to the strength of the interaction between the imidazole-fused porphyrin core and the acceptor cyanoacrylic or carboxylic anchoring group. Dye **37** shows the lowest gap among the series (1.98 vs. 2.01 eV for **38** and 2.02 eV for **39**) and due to the cyanoacrylic anchoring group, when used as a sensitizer, generates the best performing device (PCE = 3.67%), in accordance to the high *J*_*sc*_ (7.41 mAcm^−2^) and the broadened IPCE curve.

The combination of more than one fused moiety as in **40**-**41** can further enhance the photovoltaic properties (Figure 14D) (Hayashi et al., 2013). The fused quinoxaline moiety in **40** has an electron-acceptor character and the addition of a triarylamino electron donor at the opposite β,β'-edge through a fused imidazole moiety (**41**) allows a broadening and a red-shift of the Soret and Q bands with enhanced light harvesting. In agreement, the electrochemical data show a negative shift of the first oxidation potential of **41** in comparison to **40** (0.92 V vs. NHE and 0.98 V vs. NHE, respectively, Table S1), while the first reduction potentials are almost the same for the two compounds (−1.11 V vs. NHE and −1.13 V vs. NHE, respectively, Table S1). The concomitant introduction of a triarylamino and fused imidazole moieties has a more significant effect on the HOMO energy than on the LUMO, with a lowering of the electrochemical HOMO-LUMO gap (2.03 eV for **41** and 2.11 eV for **40**). Moreover, when **41** is used to sensitized TiO_2_, a flat and intense IPCE spectrum is recorded (up to 80%) producing a remarkable PCE (6.8%).

**Figure 15:**
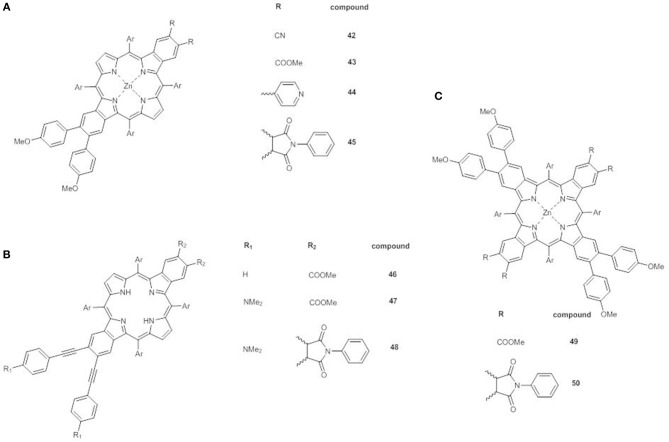
**(A)** β-functionalized dibenzoporphyrins **42-45**; **(B)** β-functionalized dibenzoporphyrins with ethynylphenyl bridges **46-48**; **(C)** β-functionalized tetrabenzoporphyrins **49-50**.

A very promising recent approach involves the synthesis of β-functionalized push-pull *opp*-dibenzoporphyrins. In these structures two benzene units are fused at the opposite β,β'-positions of the core, and carry at one end an electron donor methoxyphenyl group and at the other a variety of electron acceptor substituents. The donor group can be linked to the benzene ring by a phenyl bridge (**42**-**45)** (Figure 15A) (Jinadasa et al., 2015) or by a more conjugated ethynylphenyl spacer (**46**-**48)** (Figure 15B) (Jinadasa et al., 2017).

The overall architecture appears therefore as an extended π-conjugated push-pull system, suitable for application in optoelectronics or photovoltaics after proper chemical functionalization.

The electronic absorption spectra of **42-45** display some peculiarities: (a) the B bands have a shoulder; (b) an additional absorption in the range 380–405 nm appears; (c) extra Q bands at 600–650 nm are present. Moreover, in accordance to the strong electron acceptor properties of the substituents, these effects are more pronounced for compounds **42** and **45**.

Indeed, for example, while the replacement of vicinal esters in **43** with cyclic imide in **45** has no effect on the first oxidation potentials (Table S1), the introduction of strong electron-withdrawing cyano groups in **42** causes a positive shift of the anodic potential.

DFT calculation have shown that the electron density of the HOMO spans over the porphyrin core and the two fused benzene rings, with only a minimal involvement of the methoxyphenyl donor group, due to the hampered rotation of the benzene rings around the C-C single bond. On the other hand, the LUMO orbital is mainly on the porphyrin core and appears affected by the introduction of strong electron acceptor moieties. Therefore, the HOMO-LUMO gap and the push-pull character of **42-45** can be finely tuned.

The insertion of an ethynyl spacer between the porphyrin core and the phenyl moiety carrying the donor group assures a better conjugation of the core with the electron donor, thus enhancing the push-pull effect, as in **46-48**. Indeed the UV-Vis spectrum of **46** shows a red shift of the Soret band in comparison to that of **43** as free base.

Moreover, when the *opp*-dibenzoporphyrin is endowed with a strong electron donating dimethylamino group (**47**), a huge modification of the absorption pattern occurs with respect to **46**. A shoulder at lower wavelengths appears near the B band, which is also more broadened and bathochromically shifted. A similar red shift occurs also for the Q bands, with a significant increase in intensity of that at 537 nm. All these effects are produced by the simple addition of the electron donor, highlighting therefore its unique role.

The replacement of the two carboxymethyl acceptors with a cyclic imide in **48** enhances further increases the red shift of the Soret and the Q bands, and also the intensity of the Q band at 584 nm.

As expected on the basis of the electronic absorption data, the HOMO and LUMO orbitals in **46** are mainly on the porphyrin core, the two fused benzene rings and the ethynyl spacer. By introduction of the dimethylamino group in **47** the HOMO orbital shifts mostly on the donor moiety, while the LUMO remains on the porphyrin core, without any involvement of the carboxymethyl groups. On the contrary, in the presence of the cyclic imide (**48**), the electron density of the LUMO moves toward the acceptor, with a decrease of the electrochemical HOMO-LUMO energy gap (1.94 vs. 2.41 eV for **46** and 2.01 eV for **47**, Table S1) and the generation of the most performing push-pull system.

β-functionalized *trans*-A_2_B_2_ push-pull tetrabenzoporphyrins **49** and **50** (Figure 15C) (Kumar et al., 2018) represent the latest evolution of the concept of *opp*-dibenzoporphyrins. The large extended π-delocalization gives them distinctive physicochemical and photophysical properties, making them interesting candidates for DSSCs even if their synthesis is quite tedious.

The fusion of two more benzene rings on the free pyrrole units of an *opp*-dibenzoporphyrin causes substantial variations in the UV-Vis pattern. **49** shows a broad and red shifted B band at 488 nm, which is further red shifted to 507 nm by substitution of the carboxymethyl acceptor units with cyclic imides. More notably the Q bands of **49** appear overlapped and quite intense over a significant range of wavelengths (570–720 nm), while for **50** a very intense Q band at 721 nm is observed, accompanied by a weak absorption at lower wavelength (649 nm).

DFT calculations have evidenced for both **49** and **50** a HOMO located on the porphyrin core and on the four benzene units, while the LUMO spans the core, the two benzene moieties carrying the electron acceptor group and for **50** the cyclic imides, leaving untouched the other two benzenes with the methoxyphenyl groups.

The energy level diagram for **49** and **50** shows a remarkable deviation from the Gouterman model, with a significant splitting of the two almost degenerate HOMOs. The presence of the electron withdrawing cyclic imide stabilizes both the LUMO and the HOMO energy levels, so that the both the calculated and the electrochemical HOMO-LUMO gaps for **50** are lower than that of **49** (2.14 vs. 2.26 eV and 1.73 vs. 1.97 V, respectively, Table S1).

Therefore, the presence of two electron donor and two electron acceptor moieties in β-pyrrolic position of Zn^II^ tetrabenzoporphyrins promotes huge and tunable effects on their electronic properties, which make these systems promising for application in DSSCs.

A key point that has to be underlined is that possible modifications of the electronic properties of the porphyrin can occur after the chemisorption on the semiconductor surface due to strong intermolecular dye/TiO_2_ or dye/dye interactions, and to scattering.

For example, a recent investigation (Munir et al., 2015) on 5, 10, 15, 20-tetraphenylsulphonic porphyrin has shown a bathochromic shift of the B band and a lowering of the number of the Q bands from four to two after chemisorption on TiO_2_. The interaction of the free porphyrin core with TiO_2_ is strongly favored by the presence of the sulphonic acid groups, which allow a flattening of the macrocycle on the semiconductor surface and an interaction of the titanium ions with the central cavity of the porphyrin.

For dye SM315 a slight blue shift of the bands has been reported on TiO_2_, consistent with a lowering of the molecular dipole moment due to the deprotonation of the carboxylic group after anchoring (Mathew et al., 2014).

In other papers (Liang et al., 2015; Panagiotakis et al., 2018) a remarkable broadening and a red-shift of the electronic absorption bands of ZnII porphyrins is reported after chemisorption on TiO_2_, with an enhanced light harvesting in the NIR range of wavelengths. Red-shifting of thee Q bands is also evident in the porphyrin **13**, endowed with DTE unit in β-position, upon being adsorbed on TiO_2_ photoanode (Di Carlo et al., 2017).

On the other hand, when compounds **7** and **10** (Figure 4) are anchored on the surface of TiO_2_, the electronic absorption spectra display the same number of bands than those of the dyes in solution, without any significant difference in the wavelength of the absorption maxima. The only effect of chemisorption is a faint broadening of the bands due to scattering (Di Carlo et al., 2014b).

Therefore, a good experimental practice suggests to record the UV-Vis spectra of the dyes not only in solution, but also once absorbed on the semiconductor.

## Conclusions

The purpose of this mini-review is to survey the more effective strategies used to tune the light harvesting ability of porphyrin chromophores for photovoltaic application in DSSCs. Particular attention is devoted to porphyrin dyes showing A_4_ β-pyrrolic substitution pattern which have been less extensively studied than the *trans*-A_2_BC push-pull counterparts despite their promising results in TiO_2_ sensitization. The *meso*-architecture, which relies on a push-pull system arranged on 5,15 *meso* positions of a porphyrin core, exhibits a stronger charge-transfer character compared with the relative A_4_-porphyrins bearing the donor-acceptor system through the pyrrolic positions. This feature strongly affects the electrochemical properties of the dyes impacting the HOMO level, LUMO level and the resulting HOMO-LUMO energy gap thus influencing both the dynamics of electron transfer processes and the sunlight capture. Indeed, while *trans*-A_2_BC push-pull porphyrins show intense light absorption in the visible region up to the NIR but leaving uncovered the visible portion between Soret and Q bands, the A_4_ β-porphyrins are stronger light harvesters in the entire visible spectrum and their photon collection in the NIR is less significant. Since several tailoring strategies have been investigated to tune the light absorption profile of *meso*-porphyrins, some of them have been adopted as well to improve the light harvesting ability of A_4_ β-porphyrin dyes. The majority deals with the narrowing of the HOMO-LUMO gap in order to promote broadening and red-shifting of absorption bands with the specific aim to boost photon collection both in the visible and near infrared regions. The most advances on designing effective β-porphyrin dyes range from the coupling of porphyrin core with conjugated strong acceptors, which stabilize the LUMO level and simultaneously serve as additional chromophores turning the porphyrin into panchromatic dyes, to rising the HOMO level by adding electron-donating moieties on the periphery of the porphyrin core. Another exotic but effective strategy to extend the photon collection up to the NIR is the extension of π-conjugation as a result of coupling two or more porphyrin units either through β-positioned linear linkers or fusing the cores by means of pyrrolic substituents. A_4_ β-porphyrin dyes due to the less demanding synthetic pathways and the remarkable PCEs are considered very promising to pass from the lab- to the mass scale production, however to date few strategies have been investigated on a judicious tailoring of such chromophores, thus we strongly believe that the scientific community could have plenty of room in designing new and more efficient sensitizers to boost their photovoltaic performances.

## Author Contributions

FT, GD, and AO wrote the paper and prepared the figures. MP revised the paper.

### Conflict of Interest Statement

The authors declare that the research was conducted in the absence of any commercial or financial relationships that could be construed as a potential conflict of interest.

## References

[B1] Arrechea S. Clifford J. N. Pellejà L. Aljarilla A. De La Cruz P. Palomares E. (2016). Charge recombination losses in thiophene-substituted porphyrin dye-sensitized solar cells. Dye Pigment. 126, 147–153. 10.1016/j.dyepig.2015.11.002

[B2] Barea E. M. Caballero R. López-Arroyo L. Guerrero A. de la Cruz P. Langa F.. (2011). Triplication of the photocurrent in dye solar cells by increasing the elongation of the π-conjugation in Zn-porphyrin sensitizers. ChemPhysChem 12, 961–965. 10.1002/cphc.201000958 21381177

[B3] Becke A. D. (1993). Density-functional thermochemistry.III. The role of exact change. J. Phys. Chem. 98, 5648–5652. 10.1063/1.464913

[B4] Bodedla G. B. Wang H. Chang S. Chen S. Chen T. Zhao J. (2018). β-functionalized imidazole-fused porphyrin-donor-based dyes: effect of π-linker and acceptor on optoelectronic and photovoltaic properties. ChemistrySelect 3, 2558–2564. 10.1002/slct.201702652

[B5] Campbell W. M. Burrell A. K. Officer D. L. Jolley K. W. (2004). Porphyrins as light harvesters in the dye-sensitized TiO2 solar cell. Coord. Chem. Rev. 248, 1363–1379. 10.1016/j.ccr.2004.01.007

[B6] Chang Y.-C. Wang C.-L. Pan T.-Y. Hong S.-H. Lan C.-M. Kuo H.-H.. (2011). A strategy to design highly efficient porphyrin sensitizers for dye-sensitized solar cells. Chem. Commun. 47, 8910–8912. 10.1039/c1cc12764k 21677971

[B7] Charisiadis A. Stangel C. Nikolaou V. Roy M. S. Sharma G. D. Coutsolelos A. G. (2015). Supramolecular assembling of zinc porphyrin with a π-conjugated oligo(phenylenevinylene) (OPPV) molecular wire for dye sensitized solar cell. RSC Adv. 5, 88508–88519. 10.1039/C5RA16394C

[B8] Chen J. Li K.-L. Guo Y. Liu C. Guo C.-C. Chen Q.-Y. (2013). Design and synthesis of β-multi-substituted push-pull porphyrins. RSC Adv. 3, 8227–8231. 10.1039/c3ra40308d

[B9] Cheng H. L. Huang Z. S. Wang L. Meier H. Cao D. (2017). Synthesis and photovoltaic performance of the porphyrin based sensitizers with 2H-[1,2,3]Triazolo[4,5-c]pyridine and benzotriazole as auxiliary acceptors. Dye. Pigment. 137, 143–151. 10.1016/j.dyepig.2016.10.006

[B10] Clifford J. N. Martínez-Ferrero E. Viterisi A. Palomares E. (2011). Sensitizer molecular structure-device efficiency relationship in dye sensitized solar cells. Chem. Soc. Rev. 40, 1635–1646. 10.1039/B920664G 21076736

[B11] Cossi M. Rega N. Scalmani G. Barone V. (2003). Energies, structures, and electronic properties of molecules in solution with the C-PCM solvation model. J. Comput. Chem. 24, 669–681. 10.1002/jcc.10189 12666158

[B12] Covezzi A. Orbelli Biroli A. Tessore F. Forni A. Marinotto D. Biagini P. (2016). 4D-π-1A Type β-substituted ZnII-porphyrins: ideal green sensitizers for building-integrated photovoltaics. Chem. Commun. 85, 12642–12645. 10.1039/C6CC05870A 27722549

[B13] Curtiss L. A. McGrath M. P. Blaudeau J. –P. Davis N. E. Binning R. C. Jr. Radom L. (1995). Extension of Gaussian-2 theory to molecules containing third-row atoms Ga-Kr. J. Chem. Phys. 103, 6140–6113. 10.1063/1.470438

[B14] De Angelis F. Fantacci S. Sgamellotti A. Pizzotti M. Tessore F. Orbelli Biroli A. (2007). Time-dependent and coupled-perturbed DFT and HF investigations on the absorption spectrum and non-linear optical properties of push-pull M(II)-porphyrin complexes (M = Zn, Cu, Ni.). Chem. Phys. Lett. 447, 10–15. 10.1016/j.cplett.2007.08.096

[B15] Di Carlo G. Caramori S. Casarin L. Orbelli Biroli A. Tessore F. Argazzi R. (2017). Charge transfer dynamics in β and meso substituted dithienylethylene porphyrins. J. Phys. Chem. C 121, 18385–18400. 10.1021/acs.jpcc.7b05823

[B16] Di Carlo G. Caramori S. Trifiletti V. Giannuzzi R. De Marco L. Pizzotti M. (2014b). Influence of the porphyrinic structure on electron transfer processes at the electrolyte/dye/TiO2 interface in PSSCs: a comparison between meso push-pull and β-pyrrolic architectures. ACS Appl. Mater. Interf. 6, 15841–15852. 10.1021/am503113x 25089649

[B17] Di Carlo G. Orbelli Biroli A. Pizzotti M. Tessore F. Trifiletti V. Ruffo R.. (2013). Tetraaryl ZnII porphyrinates substituted at β-pyrrolic positions as sensitizers in dye-sensitized solar cells: a comparison with meso-disubstituted push-pull znii porphyrinates. Chem. A Eur. J. 19, 10723–10740. 10.1002/chem.201300219 23794212

[B18] Di Carlo G. Orbelli Biroli A. Tessore F. Caramori S. Pizzotti M. (2018). β-substituted ZnII porphyrins as dyes for DSSC: a possible approach to photovoltaic windows. Coord. Chem. Rev. 358, 153–177. 10.1016/j.ccr.2017.12.012

[B19] Di Carlo G. Orbelli Biroli A. Tessore F. Pizzotti M. Mussini P. R. Amat A. (2014a). Physicochemical Investigation of the panchromatic effect on β-substituted Zn II porphyrinates for DSSCs: the role of the π bridge between a dithienylethylene unit and the porphyrinic ring. J. Phys. Chem. C 118, 7307–7320. 10.1021/jp412087f

[B20] Di Carlo G. Orbelli Biroli A. Tessore F. Rizzato S. Forni A. Magnano G. (2015). Light-induced regiospecific bromination of meso-tetra(3,5-di-tert-butylphenyl)porphyrin on 2,12 β-pyrrolic position. J. Org. Chem. 80, 4973–7980. 10.1021/acs.joc.5b00367 25894251

[B21] Freitag M. Teuscher J. Saygili Y. Zhang X. Girodano F. Liska P. (2017). Dye-sensitized solar cells for efficient power generation under ambient lighting. Nat. Photon. 11, 372–379. 10.1038/nphoton.2017.60

[B22] Frisch M. J. Trucks G. W. Schlegel H. B. Scuseria G. E. Robb M. A. Cheeseman J. R. (2004). Gaussian 03 Wallingford, CT: Gaussian, Inc.

[B23] Frisch M. J. Trucks G. W. Schlegel H. B. Scuseria G. E. Robb M. A. Cheeseman J. R. (2009). Gaussian 09 Wallingford, CT: Gaussian, Inc.

[B24] Giovannetti R. (2014). The Use of Spectrophotometry UV-Vis for the Study of Porphyrins. Macro to Nanospectroscopy Jamal Uddin, IntechOpen. Available online at: https://www.intechopen.com/books/macro-to-nano-spectroscopy/the-use-of-spectrophotometry-uv-vis-for-the-study-of-porphyrins (accessed June 29, 2012).

[B25] Gong J. Sumathy K. Qiao Q. Zhou Z. (2017). Review on dye-sensitized solar cells (DSSCs): advanced techniques and research trends. Ren. Sust. En. Rev. 68, 234–246. 10.1016/j.rser.2016.09.097

[B26] Gouterman M. (1961). Spectra of porphyrins. J. Mol. Spectr. 6, 138–163.

[B27] Gouterman M. (1978). Optical spectra and electronic structure of porphyrins and related rings, in Porphyrins 3, ed Dolphin D. (New York, NY: Academic Press), 1–165.

[B28] Griffith M. J. Sunahara K. Wagner P. Wagner K. Wallace G. G. Officer D. L. (2012). Porphyrins for dye-sensitized solar cells: new insights into efficiency-determining electron transfer steps. Chem. Commun. 48, 4145–4162. 10.1039/c2cc30677h 22441329

[B29] Gritzner G. (1990). Polarographic half-wave potentials of cations in nonaqueous solvents. Pure Appl. Chem. 62, 1839–1858. 10.1351/pac199062091839

[B30] Gritzner G. Köta J. (1984). Recommendations on reporting electrode potentials in nonaqueous solvents: IUPC commission on electrochemistry. Electrochim. Acta 29, 869–873. 10.1016/0013-4686(84)80027-4

[B31] Hagfeldt A. Boschloo G. Sun L. Kloo L. Pettersson H. (2010). Dye-sensitized solar cells. Chem. Rev. 110, 6595–6663. 10.1021/cr900356p 20831177

[B32] Hayashi H. Touchy A. S. Kinjo Y. Kurotobi K. Toude Y. Ito S.. (2013). Triarylamine-substituted imidazole- and quinoxaline-fused push-pull porphyrins for dye-sensitized solar cells. ChemSusChem 6, 508–517. 10.1002/cssc.201200869 23401121

[B33] Higashino T. Imahori H. (2015). Porphyrins as excellent dyes for dye-sensitized solar cells: recent developments and insights. Dalton Trans. 44, 448–463. 10.1039/C4DT02756F 25381701

[B34] Hsieh C. Lu H. Chiu C. Lee C. Chuang S. Mai C. (2010). Synthesis and characterization of porphyrin sensitizers with various electron-donating substituents for highly efficient dye-sensitized solar cells. J. Mater. Chem. 20, 1127–1134. 10.1039/B919645E

[B35] Huang C.-Y. Hsu C.-Y. Yang L.-Y. Lee C.-J. Yang T.-F. Hsu C.-C. (2012). A systematic study of electrochemical and spectral properties for the electronic interactions in porphyrin-triphenylamine conjugates. Eur. J. Inorg. Chem. 7, 1038–1047. 10.1002/ejic.201101033

[B36] Jinadasa R. G. W. Fang Y. Kumar S. Osinski A. J. Jiang X. Ziegler C.. (2015). β-functionalized push-pull opp-dibenzoporphyrins. J. Org. Chem. 80, 12076–12087. 10.1021/acs.joc.5b01906 26580715

[B37] Jinadasa R. G. W. Thomas M. B. D' Souza F. Wang H. (2017). Investigation of the push-pull effects on β-functionalized benzoporphyrins bearing an ethynylphenyl bridge. Phys. Chem. Chem. Phys. 19, 13182–13188. 10.1039/C7CP00024C 28489116

[B38] Kadish K. M. Van Caemelbecke E. (2003). Electrochemistry of porphyrins and related macrocycles. J. Solid State Electrochem. 7, 254–258. 10.1007/s10008-002-0306-3

[B39] Kakiage K. Aoyama Y. Tano T. Oya K. Fujisawa J. Hanaya M. (2015). Highly-efficient dye-sensitized solar cells with collaborative sensitization by silyl-anchor and carboxy-anchor dyes. Chem. Commun. 51, 15894–15897. 10.1039/C5CC06759F 26393334

[B40] Kao M. C. Chen H. Z. Young S.-L. Kung C. Y. Lin C. C. (2009). The effects of the thickness of TiO2 films on the performance of dye-sensitized solar cells. Thin Solid Films 517, 5096–5099. 10.1016/j.tsf.2009.03.102

[B41] Kumar S. Jiang X. Shan W. Jiandasa R. G. W. Kadish K. M. Wang H. (2018). β-functionalized trans-A2B2 push-pull tetrabenzoporphyrins. Chem. Commun. 54, 5303–5306. 10.1039/C7CC09743C 29503998

[B42] Lee C.-W. Lu H.-P. Lan C.-M. Huang Y.-L. Liang Y.-R. Yen W.-N.. (2009). Novel zinc porphyrin sensitizers for dye-sensitized solar cells: synthesis and spectral, electrochemical, and photovoltaic properties. Chem. A Eur. J. 15, 1403–1412. 10.1002/chem.200801572 19097125

[B43] Li C. Luo L. Wu D. Jiang R. Lan J. Wang R. (2016). Porphyrins with intense absorptivity: highly efficient sensitizers with a photovoltaic efficiency of up to 10.7% without a cosensitizer and a coabsorbate. J. Mater. Chem. A 4, 11829–11834. 10.1039/C6TA02888H

[B44] Li L.-L. Diau E. W.-G. (2013). Porphyrin-sensitized solar cells. Chem. Soc. Rev. 42, 291–304. 10.1039/C2CS35257E 23023240

[B45] Liang Y. Xue X. Zhang W. Fan C; Li, Y. Zhang B. (2015). Novel D-π-A structured porphyrin dyes containing various diarylamino moieties for dye-sensitized solar cells. Dyes Pigm. 115, 7–16. 10.1016/j.dyepig.2014.12.006

[B46] Liu J. Yang X. Sun L. (2013). Axial anchoring designed silicon-porphyrin sensitizers for efficient dye-sensitized solar cells. Chem. Commun. 49, 11785–11787. 10.1039/c3cc46581k 24213560

[B47] Llanos J. Brito I. Espinoza D. Sekar R. Manidurai P. (2018). A down-shifting Eu3+-doped Y2wo6/Tio2 photoelectrode for improved light harvesting in dye-sensitized solar cells. R. Soc. Open Sci. 5, 171054–171062. 10.1098/rsos.171054 29515831 PMC5830720

[B48] Lu F. Feng Y. Wang X. Zhao Y. Yang G. Zhang J. (2017). Influence of the additional electron-withdrawing unit in β-functionalized porphyrin sensitizers on the photovoltaic performance of dye-sensitized solar cells. Dye. Pigment. 139, 255–263. 10.1016/j.dyepig.2016.12.027

[B49] Lu F. Wang X. Zhao Y. Yang G. Zhang J. Zhang B. (2016). Studies on D-A-π-a structured porphyrin sensitizers with different additional electron-withdrawing unit. J. Power Sources 333, 1–9. 10.1016/j.jpowsour.2016.09.149

[B50] Lu J. Liu S. Wang M. (2018). Push-pull porphyrins as light-harvesters for efficient dye-sensitized solar cells. Front. Chem. 6:541. 10.3389/fchem.2018.00541 30519554 PMC6251255

[B51] Luo J. Xu M. Li R. Huang K.-W. Jiang C. Qi Q.. (2014). N -Annulated perylene as an efficient electron donor for porphyrin-based dyes: enhanced light-harvesting ability and high-efficiency CO(II/III)-based dye-sensitized solar cells. J. Am. Chem. Soc. 136, 265–272. 10.1021/ja409291g 24345083

[B52] Magnano G. Marinotto D. Cipolla M. P. Trifiletti V. Listorti A. Mussini P. R.. (2016). Influence of alkoxy chain envelopes on the interfacial photoinduced processes in tetraarylporphyrin-sensitized solar cells. Phys. Chem. Chem. Phys. 18, 9577–9585. 10.1039/C6CP00129G 26987742

[B53] Mathew S. Astani N. A. Curchod B. F. E. Delcamp J. H. Marszalek M. Frey J. (2016). Synthesis, characterization and *ab initio* investigation of a panchromatic ullazine-porphyrin photosensitizer for dye-sensitized solar cells. J. Mater. Chem. A 4, 2332–2339. 10.1039/C5TA08728G

[B54] Mathew S. Yella A. Gao P. Humphry-Baker R. Curchod B. F. E. Ashari-Astani N.. (2014). Dye-sensitized solar cells with 13% efficiency achieved through the molecular engineering of porphyrin sensitizers. Nat. Chem. 6, 242–247. 10.1038/nchem.1861 24557140

[B55] Munir S. Shah S. M. Hussain H. Siddiq M. (2015). Adsorption of porphyrin and carminic acid on TiO2 nanoparticles: a photo-active nano-hybrid material for hybrid bulk heterojunction. J. Phothochem. Photobiol. B 153, 397–404. 10.1016/j.jphotobiol.2015.10.029 26555643

[B56] Mussini P. R. Orbelli Biroli A. Tessore F. Pizzotti M. Biaggi C. Di Carlo G. (2012). Modulating the electronic properties of asymmetric push-pull and symmetric Zn(II)-diarylporphyrinates with para substituted phenylethynyl moieties in 5,15 meso position: a combined electrochemical and spectroscopic investigation. Electrochim. Acta 85, 509–523. 10.1016/j.electacta.2012.08.039

[B57] Nazeeruddin M. K. de Angelis F. Fantacci S. Selloni A. Viscardi G. Liska P.. (2005). Combined experimental and DFT-TDDFT computational study of photoelectrochemical cell ruthenium sensitizers. J. Am. Chem. Soc. 127, 16835–16847. 10.1021/ja052467l 16316230

[B58] Nazeeruddin M. K. Kay A. Rodicio I. Humphry-Baker R. Müller E. Liska P. (1993). Conversion of light to electricity by cis-X2-bis((2,2'-bipyridyl-4,4'-dicarboxilate)ruthenium(II) charge-transfer sensitizers (X = CL–, BR–, I–, CN–, and SCN–) on nanocrystalline TiO_2_ electrodes. J. Am. Chem. Soc. 115, 6382–6390.

[B59] Obraztsov I. Kutner W. D' Souza F. (2017). Evolution of molecular design of porphyrin chromophores for photovoltaic materials of superior light-to-electricity conversion efficiency. Sol. RRL 1, 1600002–1600016. 10.1002/solr.201600002

[B60] Orbelli Biroli A. Tessore F. Pizzotti M. Biaggi C. Ugo R. Caramori S. (2011). A multitechnique physicochemical investigation of various factors controlling the photoaction spectra and of some aspects of the electron transfer for a series of push-pull Zn(II) porphyrins acting as dyes in DSSCs. J. Phys. Chem. C 115, 23170–23182. 10.1021/jp2030363

[B61] Orbelli Biroli A. Tessore F. Righetto S. Forni A. Macchioni A. Rocchigiani L. (2017). Intriguing influence of –COOH-driven intermolecular aggregation and acid-base interactions with DMF orn the second order NLO response of 5,15 push-pull diaryl ZnII prophyrinates. Inorg. Chem. 56, 6438–6450. 10.1021/acs.inorgchem.7b00510 28475347

[B62] Orbelli Biroli A. Tessore F. Vece V. Di Carlo G. Mussini P. R. Trifiletti V. (2015). Highly improved performance of ZnII tetraarylporphyrinates in DSSCs by the presence of octyloxy chains in the aryl rings. J. Mater. Chem. A 6, 2954–2959. 10.1039/C4TA05233A

[B63] O'Regan B. Grätzel M. (1991). A low-cost high-efficiency solar cell based on colloidal TiO2 films. Nature 353, 737–740.

[B64] Pan B. Zhu Y. Z. Ye D. Zheng J. Y. (2018). Improved Conversion efficiency in dye-sensitized solar cells based on porphyrin dyes with dithieno[3,2-b:2′,3′-d]pyrrole donor. Dye. Pigment. 150, 223–230. 10.1016/j.dyepig.2017.12.018

[B65] Pan J. Song H. Lian C. Liu H. Xie Y. (2017). Cocktail co-sensitization of porphyrin dyes with additional donors and acceptors for developing efficient dye-sensitized solar cells. Dye. Pigment. 140, 36–46. 10.1016/j.dyepig.2017.01.027

[B66] Panagiotakis S. Giannoudis E. Charisiadis A. Paravatou R. Lazaridi M.-E. Kandyli M. (2018). Increased efficiency of dye-sensitized solar cells by incorporation of a π spacer in donor-acceptor zinc porphyrins bearing a cyanoacrylic acid as an anchoring group. Eur. J. Inorg. Chem. 2369–2379. 10.1002/ejic.201800123

[B67] Parsa Z. Naghavi S. S. Safari N. (2018). Designing push–pull porphyrins for efficient dye-sensitized solar cells. J. Phys. Chem. A 122, 5870–5877. 10.1021/acs.jpca.8b03668 29921128

[B68] Pellejà L. Kumar C. V. Clifford J. N. Palomares E. (2014). D-π-A porphyrin employing an indoline donor group for high efficiency dye-sensitized solar cells. J. Phys. Chem. C 118, 16504–16509. 10.1021/jp411715n

[B69] Possanza F. Limosani F. Tagliatesta P. Zanoni R. Scarselli M. Ciotta E.. (2018). Functionalization of carbon spheres with a porphyrin–ferrocene dyad. ChemPhysChem 19, 2243–2249. 10.1002/cphc.201800277 29781239

[B70] Rahman M. M. Ko M. J. Lee J.-J. (2015). Novel energy relay dyes for high efficiency dye-sensitized solar cells. Nanoscale 7, 3526–3531. 10.1039/C4NR06645F 25630367

[B71] Song H. Liu Q. Xie Y. (2018). Porphyrin-sensitized solar cells: systematic molecular optimization, coadsorption and cosensitization. Chem. Commun. 54, 1811–1824. 10.1039/C7CC09671B 29372729

[B72] Sunahara K. Furube A. Katoh R. Mori S. Griffith M. J. Wallace G. G. (2011). Coexistence of femtosecond- and nonelectron-injecting dyes in dye-sensitized solar cells: inhomogeniety limits the efficiency. J. Phys. Chem. C 115, 22084–22088. 10.1021/jp2093109

[B73] Walsh P. J. Gordon K. C. Officer D. L. Campbell W. M. (2006). A DFT study of the optical properties of substituted Zn(II)TPP complexes. J. Mol. Struct. Theochem. 759, 17–24. 10.1016/j.theochem.2005.10.049

[B74] Wang C.-L. Chang Y.-C. Lan C.-M. Lo C.-F. Wei-Guang Diau E. Lin C.-Y. (2011). Enhanced light harvesting with π-conjugated cyclic aromatic hydrocarbons for porphyrin-sensitized solar cells. Energy Environ. Sci. 4, 1788–1795. 10.1039/c0ee00767f

[B75] Wang Y. Chen B. Wu W. Li X. Zhu W. Tian H.. (2014). Efficient solar cells sensitized by porphyrins with an extended conjugation framework and a carbazole donor: from molecular design to cosensitization. Angew. Chem. 53, 10779–10783. 10.1002/anie.201406190 25132108

[B76] Wang Y. Li X. Liu B. Wu W. Zhu W. Xie Y. (2013). Porphyrins bearing long alkoxyl chains and carbazole for dye-sensitized solar cells: tuning cell performance through an ethynylene bridge. RSC Adv. 34, 14780–14790. 10.1039/c3ra40788h

[B77] Wu C.-H. Pan T.-Y. Hong S.-H. Wang C.-L. Kuo H.-H. Chu Y.-Y.. (2012). A fluorene-modified porphyrin for efficient dye-sensitized solar cells. Chem. Commun. 48, 4329–4331. 10.1039/c2cc30892d 22446840

[B78] Xie Y. Tang Y. Wu W. Wang Y. Liu J. Li X.. (2015). Porphyrin cosensitization for a photovoltaic efficiency of 11.5%: a record for non-ruthenium solar cells based on iodine electrolyte. J. Am. Chem. Soc. 137, 14055–14058. 10.1021/jacs.5b09665 26492075

[B79] Yella A. Lee H.-W. Tsao H. N. Yi C. Chandiran A. K. Nazeeruddin M. K.. (2011). Porphyrin-sensitized solar cells with cobalt (II/III)-based redox electrolyte exceed 12 percent efficiency. Science 334, 629–634. 10.1126/science.1209688 22053043

[B80] Yella A. Mai C.-L. Zakeeruddin S. M. Chang S. -N. Hsieh C.-H. Yeh C. -Y. (2014). Molecular engineering of push-pull porphyrins for highly efficient dye-sensitized solar cells: the role of benzene spacers. Angew. Chem. 53, 2973–2977. 10.1002/anie.201309343 24501108

[B81] Yu Q. Wang Y. Yi Z. Zhang J. Zhang M. Wang P.. (2010). High-efficiency dye-sensitized solar cells: the influence of lithium ions on exciton dissociation, charge recombination, and surface states. ACS NANO 4, 6032–6038. 10.1021/nn101384e 20923204

[B82] Yum J. –H. Baranoff E. Wenger S. Nazeeruddin M. K. Grätzel M. (2011). Panchromatic engineering for dye-sensitized solar cells. Energy Environ. Sci. 4, 842–857. 10.1039/C0EE00536C

