# Supplementary material for: Efficient Sunlight Harvesting by A4 β-Pyrrolic Substituted ZnII Porphyrins: A Mini-Review
Source: Front Chem. 2019 Apr 11;7:177. doi: 10.3389/fchem.2019.00177 (PMC6470396; doi:10.3389/fchem.2019.00177)
Supplement: Supplementary file 1 [file Table_1.DOCX]

**Table S1. Electrochemical data derived from cyclic voltammetry measurements and DFT computational data for the A4 β-pyrrolic Zn^II^ porphyrins discussed in the present mini-review** (for the experimental details the reader is addressed to the references cited in the main text).

| **compound** | **E^0’^_Ic_**  **(V)** | **E^0’^_IIc_**  **(V)** | **E^0’^_Ia_**  **(V)** | **E^0’^_IIa_**  **(V)** | **L**  **(eV)** | **H**  **(eV)** | **E_g,_*_EC_***  **(eV)** | **L**  **(eV)** | **H**  **(eV)** | **E_g,_*_DFT_***  **(eV)** |
| --- | --- | --- | --- | --- | --- | --- | --- | --- | --- | --- |
| **4** | -1.738 | -2.13 | 0.382 | 0.63 | -3.06 | -5.18 | 2.12 | -2.78 | -5.37 | 2.59 |
| **5** | -1.674 | -1.92 | 0.307 | 0.46 | -3.13 | -5.11 | 1.98 | -2.83 | -5.14 | 2.30 |
| **6** | -1.752 | -2.14 | 0.339 | 0.60 | -3.05 | -5.14 | 2.09 | -2.81 | -5.38 | 2.57 |
| **7** | -1.695 | -2.10 | 0.324 | 0.63 | -3.11 | -5.12 | 2.02 | -3.08 | -5.42 | 2.33 |
| **8** | -1.655 | -2.03 | 0.298 | 0.48 | -3.15 | -5.10 | 1.95 | -3.09 | -5.15 | 2.06 |
| **2** | -1.515 | -2.07 | 0.227 | 0.36 | -3.29 | -5.03 | 1.74 | -2.96 | -4.98 | 2.02 |
| **9** | -1.56 | -2.05 | 0.17 | 0.40 | -3.24 | -4.97 | 1.73 | -3.01 | -5.00 | 1.99 |
| **10** | -1.50 | -1.89 | 0.20 | 0.44 | -3.30 | -5.00 | 1.70 | -3.18 | -5.01 | 1.84 |
| **11** | - | - | - | - | - | - | - | -2.62 | -5.23 | 2.61 |
| **12** | - | - | - | - | - | - | - | -2.83 | -5.01 | 2.18 |
| **13** | -1.70 | - | 0.66 | - | -3.10 | -5.16 | 2.06 | -2.98 | -5.25 | 2.27 |
| **14** | -1.70 | - | 0.39 | - | -3.10 | -5.19 | 2.09 | -2.87 | -5.29 | 2.42 |
| **15** | -1.70 | - | 0.33 | - | -3.10 | -5.13 | 2.02 | -2.96 | -5.24 | 2.28 |
| **17** | - | - | 0.35 | - | -3.08^a^ | -5.15 | 2.07^b^ | - | - | - |
| **18** | - | - | 0.42 | - | -3.17^a^ | -5.22 | 2.05^b^ | - | - | - |
| **19** | - | - | 0.43 | - | -3.19^a^ | -5.23 | 2.04^b^ | - | - | - |
| **20** | - | - | - | - | - | - | - | -2.69 | -4.81 | 2.12 |
| **23** | -1.62 | - | 0.14 | - | -3.18 | -4.94 | 1.77 | - | - | - |
| **24** | -1.85 | - | 0.25 | - | -2.95 | -5.05 | 2.10 | - | - | - |
| **25** | -1.83 | - | 0.28 | - | -2.97 | -5.08 | 2.11 | - | - | - |
| **26** | -1.82 | - | 0.27 | - | -2.98 | -5.07 | 2.09 | - | - | - |
| **27** | -1.82 | - | 0.28 | - | -2.97 | -5.08 | 2.11 | - | - | - |
| **28** | -1.83 | - | 0.29 | - | -2.97 | -5.09 | 2.11 | - | - | - |
| **29** | -1.85 | - | 0.29 | - | -2.95 | -5.09 | 2.14 | - | - | -- |
| **30** | -1.85 | - | 0.29 | - | -2.95 | -5.09 | 2.14 | - | - | - |
| **31** | -1.70 | - | 0.24 | - | -3.10 | -5.04 | 1.94 | -2.98 | -5.06 | 2.08 |
| **32** | -1.60 | - | 0.23 | - | -3.21 | -5.03 | 1.82 | -3.14 | -5.06 | 1.92 |
| **33** | - | - | - | - | - | - | - | -2.63 | -4.81 | 2.18 |
| **34** | -1.14^c^ | - | 0.92^c^ | - | -3.26^d^ | -5.32^d^ | 2.06 | -2.39 | -5.00 | 2.61 |
| **35** | -0.79^c^ | - | 0.85^c^ | - | -3.61^d^ | -5.25^d^ | 1.64 | -2.68 | -5.02 | 2.59 |
| **36** | -1.18^c^ | - | 0.80^c^ | - | -3.21^d^ | -5.20^d^ | 1.99 | -2.35 | -4.94 | 2.34 |
| **37** | - | - | 0.31 | - | -0.90^a,e^ | 1.08^e^ | 1.98^b^ | - | - | - |
| **38** | - | - | 0.34 | - | -0.91^a,e^ | 1.10^e^ | 2.01^b^ | - | - | - |
| **39** | - | - | 0.37 | - | -0.88^a,e^ | 1.14^e^ | 2.02^b^ | - | - | - |
| **40** | -1.13^e^ | - | 0.98^e^ | - | - | - | 2.11^e^ | -2.56 | -5.25 | 2.69 |
| **41** | -1.11^e^ | - | 0.92^e^ | - | - | - | 2.03^e^ | -2.55 | -4.81 | 2.26 |
| **42** | -1.26^c^ | - | 0.78^c^ | - | - | - | 2.04^f^ | - | - | 2.61 |
| **43** | -1.29^c^ | - | 0.72^c^ | - | - | - | 2.01^f^ | - | - | 2.60 |
| **44** | -1.42^c^ | - | 0.72^c^ | - | - | - | 2.14^f^ | - | - | 2.56 |
| **45** | -1.27^c^ | - | 0.72^c^ | - | - | - | 1.99^f^ | - | - | 2.61 |
| **46** | -1.41^g^ | - | 1.0^g^ | - | - | - | 2.41^g^ | -2.34 | -4.97 | 2.63 |
| **47** | -1.16^g^ | - | 0.84^g^ | - | - | - | 2.01^g^ | -2.18 | -4.59 | 2.41 |
| **48** | -1.10^g^ | - | 0.83^g^ | - | - | - | 1.94^g^ | -2.34 | -4.64 | 2.30 |
| **49** | -1.36^c^ | - | 0.61^c^ | - | - | - | 1.97^f^ | -2.34 | -4.60 | 2.26 |
| **50** | -1.11^c^ | - | 0.62^c^ | - | - | - | 1.73^f^ | -2.67 | -4.81 | 2.14 |

^a^ derived through eq. 5. ^b^ values of E^0-0^, derived from the intersection of the absorption and emission spectra. ^c^ values *vs* SCE. ^d^ E_LUMO_ (E_HOMO_) = -4.44 + E^0’^_Icat_ (E^0’^_Ian_). ^e^ values in V *vs* NHE. ^f^ values in V *vs* SCE. ^f^ values in V *vs* Ag/AgCl.
